# Exogenous auxin-induced ENHANCER OF SHOOT REGENERATION 2 (ESR2) enhances femaleness of cucumber by activating the *CsACS2* gene

**DOI:** 10.1093/hr/uhab085

**Published:** 2022-01-20

**Authors:** Huanhuan Niu, Hu Wang, Bosi Zhao, Jiao He, Luming Yang, Xiongfeng Ma, Jiajian Cao, Zheng Li, Junjun Shen

**Affiliations:** 1 State Key Laboratory of Crop Stress Biology in Arid Areas, College of Horticulture, Northwest A&F University, Yangling, Shaanxi 712100, China; 2 College of Horticulture, Henan Agricultural University, 63 Nongye Road, Zhengzhou 450002, China; 3 Institute of Cotton Research, Chinese Academy of Agricultural Sciences, Anyang 455000, China; 4 Zhengzhou Research Base, State Key Laboratory of Cotton Biology, Zhengzhou University, Zhengzhou 450001, China; 5 College of Horticulture, Hunan Agricultural University, Nonda Road 1, Changsha 410128, China

## Abstract

Cucumber (*Cucumis sativus* L.) has been a model for the study of sex differentiation over the last two decades. Cucumber sex differentiation is mainly under genetic control, but plant growth regulators can also influence or even change it. However, the effect of exogenous auxin application on cucumber sex differentiation is not well understood at the physiological level. In this study, we explored the effects of different exogenous auxin concentrations on cucumber varieties with different mutant sex-controlling genotypes and found that there was a dosage effect of exogenous indole-3-acetic acid (IAA) on the enhancement of cucumber femaleness. Several ACC synthetase (ACS) family members responded directly to exogenous IAA, increasing endogenous ethylene synthesis, and this process appeared to be independent of the previously identified sex-related ACC oxidase CsACO2. We further demonstrated that ENHANCER OF SHOOT REGENERATION 2 (ESR2) responded to exogenous auxin induction by binding to ERE *cis*-acting element regions in the *CsACS2* promoter, directly activating *CsACS2* expression and thus increasing endogenous ethylene content, which may induce femaleness. These findings reveal that exogenous auxin increases cucumber femaleness by inducing a sex-controlling gene and promoting ethylene synthesis.

## Introduction

Cucumber (*Cucumis sativus* L.) is an economically important vegetable crop widely cultivated throughout the world. Cucumber has become a model plant for the study of sex differentiation because of its diverse sex expression and extensive studies on its physiology and genetics [1, 2]. In terms of the ratio of different sex-type flower forms produced on the individual plant, the sexual systems of cucumber include mainly monoecy, gynoecy, hermaphroditism, andromonoecy, and androecy [3, 4].

A large number of studies have confirmed that ethylene is a core regulator of cucumber sex differentiation [5, 6]. Ethylene content has been positively correlated with the number of female flowers, whereas the application of aminoethoxyvinylglycine (AVG, which inhibits ethylene biosynthesis) and Ag^+^ reagent (which inhibits ethylene signaling) reduce plant feminization [7–10]. Three major loci (*F*/*f*, *M*/*m*, and *A*/*a*) have been proposed to determine the sex phenotype of cucumber, and all of them encode ACC synthases, the rate-limiting enzymes in ethylene synthesis [11]. Among them, *CsACS1G* is responsible for gynoecy conferred by the *F* locus [12]. The *M* gene encodes CsACS2, which is expressed in the carpel region of the female flower; its inactive form (*m*) results in the formation of bisexual flowers [13]. The *A* gene encodes CsACS11, and dysfunctional *Csacs11* (*a*) blocks the developmental pathway of female flowers, resulting in androecious plants [14]. An additional androecious mutant was identified from a mutation of the *CsACO2* gene [15]. The expression of *CsACO2* has no sex specificity, but it initiates carpel development by promoting ethylene synthesis synergistically with *CsACS1G* and *CsACS11*, both of which are expressed specifically in female flowers [12, 15]. *CsWIP1* encodes a C2H2 family transcription factor, and its mutation results in a plant with a few bisexual flowers at the lower nodes and continuous female flowers at the upper nodes [16]. *CsWIP1* is believed to arrest the development of the carpel and inhibit the expression of *CsACS2* [15, 16]. A network model has been proposed to explain the interaction among the sex-controlling genes: ethylene produced by *CsACS1G* and/or *CsACS11* suppresses the expression of the carpel suppressor *CsWIP1*, activating *CsACS2* expression to promote carpel development and inhibit stamen development [14, 17].

In addition to genetic factors and ethylene, auxin can also affect cucumber sex differentiation [6, 18–20]. Galun (1962) reported that IAA treatment could transform male flower buds into female flower buds in cucumber [11]. Takahashi & Jaffe (1984) found that exogenous IAA treatment could increase the female flower rate in cucumber by promoting ethylene biosynthesis [8]. Later, Trebitsh et al. (1987) speculated that the effect of auxin treatment on cucumber sex differentiation might be mediated by an increase in endogenous ethylene content [21].

An effect of auxin on the regulation of endogenous ethylene biosynthesis has been reported in plant growth and development. Auxin and ethylene have overlapping functions in fruit ripening [22]. A high concentration of IAA in peach (*Prunus persica* L.) fruit is necessary for ethylene biosynthesis during fruit ripening [23, 24]. In plums (*Prunus salicina* L.), exogenous auxin can promote ethylene synthesis and fruit ripening, possibly by promoting the expression of *ACS* ethylene synthesis genes and *ERF* ethylene response genes [25, 26]. In *Arabidopsis thaliana*, exogenous auxin treatment can significantly increase the expression of *AtACS4* and induce more ethylene [27]. These studies demonstrated the effect of exogenous auxin treatment on ethylene synthesis in different plants.

**Table 1:** Node numbers of female (bisexual) flowers in different cucumber lines after IAA treatment

Cucumber / IAA (mg/L)	406	406an	406a	H38	Gy14M-17
0	16.4 ± 1	0	0	2.5 ± 0.5	2.9 ± 0.7
10	16.9 ± 0.9	0.5 ± 0.5^**^	0.2 ± 0.4	2.7 ± 0.5	3.5 ± 0.5^*^
50	17.3 ± 0.7^*^	2.2 ± 0.6^**^	1.2 ± 0.4^**^	3.4 ± 0.5^**^	4.2 ± 0.8^**^
500	18.2 ± 0.8^**^	3.4 ± 0.7^**^	2.3 ± 0.8^**^	4.2 ± 0.6^**^	4.9 ± 0.7^**^

Values are mean ± SD (*n*=15 seedlings, ^*^p<0.05, ^**^p<0.01). Asterisks indicate significant differences determined by Student’s *t*-test.

The effect of auxin application on cucumber sex differentiation has been reported for more than half a century. However, whether the enhancement of cucumber femaleness under exogenous auxin treatment depends on ethylene synthesis and which regulatory factors are involved in this crosstalk still remain to be explored. In this study, we analyzed the effect of IAA on the femaleness of several cucumber varieties with different sex expressions and found that there was a dosage effect of exogenous auxin on the enhancement of femaleness. In addition, the sex-related *ACS* genes responded directly to induction by exogenous IAA, increasing endogenous ethylene synthesis. This promotion appeared to be independent of the previously identified ACC oxidase ACO2. In subsequent experiments, we demonstrated that IAA treatment-induced CsESR2 could directly activate *CsACS2* by binding to ERE *cis*-acting elements in its promoter, thereby increasing endogenous ethylene content. Therefore, this study demonstrates that exogenous auxin affects cucumber femaleness by promoting ethylene synthesis, revealing an interaction network in which ethylene acts as the core and the crosslink with the auxin pathway in cucumber sex determination.

## Results

### Exogenous auxin enhanced cucumber femaleness

**Figure 1:**
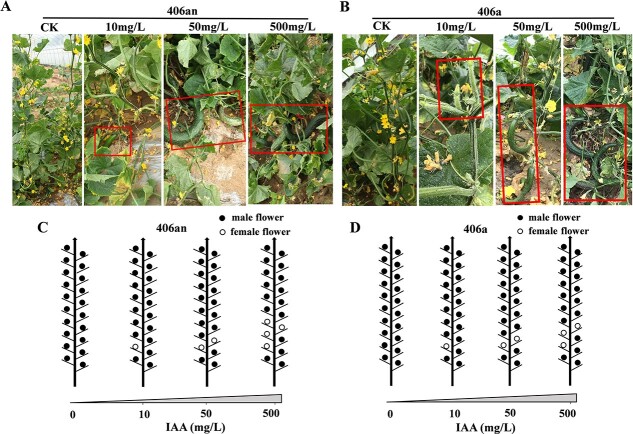
Female flowers induced by different concentrations of exogenous IAA in two cucumber androecious lines. A–B, Female flowers (in red box) of 406an and 406a induced by IAA. C–D, Schematic diagrams of the flower distribution in 406an and 406a induced by IAA. From the two-leaf stage, the seedlings were sprayed with IAA solution every five days for a total of four applications.

We first prepared four concentrations of IAA (0, 10, 50, and 500 mg/L) and selected five cucumber varieties with different sex types. After treatment with IAA solutions, the numbers of female flower nodes (or bisexual flowers in H38) were statistically analyzed. Numbers of female flowers and developing fruits increased in all tested lines after IAA treatment (Table 1). In the androecious lines 406an (Figure 1A) and 406a (Figure 1B), the female flower nodes were concentrated in the lower part of the plant, consistent with growth during the period of IAA treatment. As IAA concentration increased, the node numbers of female flowers increased to different degrees in all lines, and 406an (Figure 1C) and 406a (Figure 1D) plants showed the most obvious trend of female enhancement (Table 1). This result indicates that IAA treatment has a dosage-dependent effect on the process of femaleness induction. Most notably, in the androecious line 406an, approximately 3 and 4 female flower nodes were induced after 50 mg/L and 500 mg/L IAA treatment, respectively (Table 1). In addition, plant height was clearly inhibited at the 500 mg/L IAA concentration, indicating an obvious toxic effect (Figure S1).

**Figure 2:**
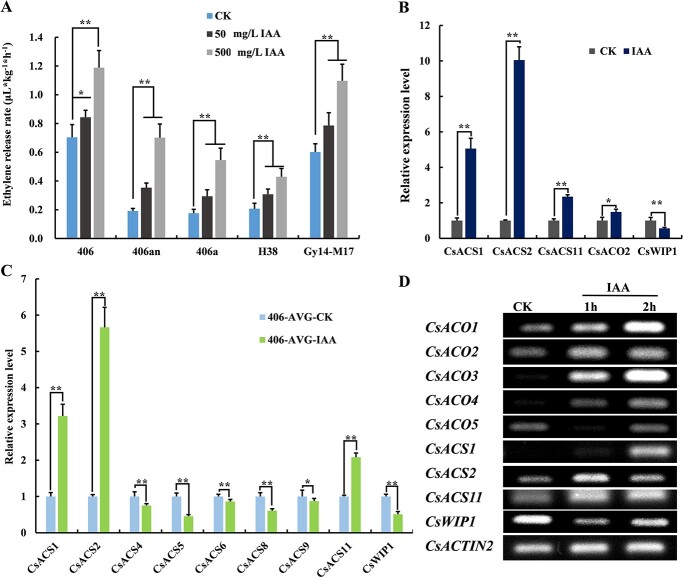
Effect of exogenous IAA treatment on ethylene-related parameters in cucumber. A, Ethylene release rate of apical buds from 5 kinds of cucumber seedlings treated with IAA solution. Five biological replicates were performed. B, Expression of sex-controlling genes in apical buds of the 406 line treated with 50 mg/L IAA for 1 h. C, Expression of all *ACS* family genes in apical buds of the 406 line treated with 50 mg/L IAA for 1 h. Seedlings at the two-leaf stage were treated with AVG for 3 days and then treated with IAA. D, Expression of all *ACO* family genes and sex-controlling genes in apical buds of 406a after 1 h and 2 h of 50 mg/L IAA treatment. *CsACTIN2* was used as the reference gene. Three biological replicates were performed in B and C. Values represent the mean and SD of all measurements, and Student’s *t*-test was used to determine significant differences in B and C (^*^p<0.05; ^**^p<0.01).

Given the femaleness enhancement observed after exogenous auxin treatment and the central role of endogenous ethylene in cucumber sex determination, we collected apical buds of different lines and measured their ethylene release rates after IAA treatment. The ethylene release rate increased significantly as IAA concentration increased in all varieties (Figure 2A). After 500 mg/L IAA treatment, the ethylene release rate of 406an showed the greatest increase, more than three times higher than that of the control. Similarly, the ethylene release rate of 406a was also increased about three-fold compared with that of the control. The increases in ethylene release rates of H38, 406, and GY14-M17 were moderate: about 100%, 80%, and 80% under the same treatment, respectively. These results showed that the change in ethylene release rate from apical buds corresponded strongly to female enhancement in different cucumber lines under exogenous auxin treatment, indicating a dosage-dependent effect of exogenous auxin on endogenous ethylene synthesis.

### Exogenous auxin induced expression of sex-controlling genes

The increases in ethylene release rate and femaleness may result from changes in the expression of ethylene synthesis-related and sex-controlling genes in cucumber. Therefore, after IAA treatment, we collected the apical buds of the 406 line and analyzed the expression of these genes (Figure 2B). *CsACS1*, *CsACS2* and *CsACS11* were induced after IAA treatment, and the expression change was greatest in *CsACS2*. The expression of *CsWIP1* was significantly inhibited after IAA treatment, and the expression level of *CsACO2* changed slightly.

Given the positive feedback effect of ethylene signaling in the process of cucumber sex determination [28], sex-controlling genes may indirectly respond to the endogenous ethylene induced by exogenous IAA. We first used the ethylene synthesis inhibitor AVG to interrupt endogenous ethylene synthesis, then treated with IAA solution. We analyzed all reported *ACS* genes in cucumber and found that only *CsACS1*, *CsACS2*, and *CsACS11* were induced by exogenous IAA directly (Figure 2C), whereas other *ACS* genes did not respond to IAA induction. Among these genes, the induction effect on *CsACS2* was the most obvious. In addition, as the suppressor of ethylene synthesis during sex determination, *CsWIP1* showed significantly inhibited expression under exogenous IAA treatment.

Although the mutation of *CsACO2* leads to an androecious line, we found that 406a still released ethylene (Figure 2A) and produced female flowers after IAA treatment (Table 1). We then analyzed the expression of all reported cucumber *ACO* genes after 1 and 2 h of IAA treatment. The expression levels of *CsACO1*, *CsACO3*, and *CsACO4* increased with increasing IAA treatment time, and this effect was most obvious for *CsACO3* (Figure 2D). *CsACS2* and *CsACS11* were induced rapidly at 1 h, and the upregulation of *CsACS2* was greater. *CsACS1* responded to auxin induction slowly and was upregulated at 2 h. The expression of *CsWIP1* was inhibited at 1 h and then restored at 2 h (Figure 2D), in contrast to the expression pattern of *CsACS2* under IAA treatment.

### Identification of regulatory factors that respond to exogenous auxin

**Figure 3:**
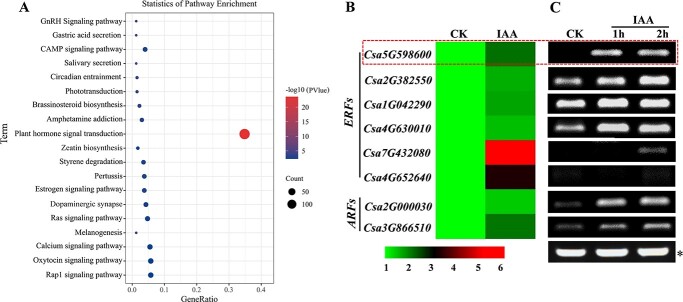
Identification of auxin-response transcription factors in the transcriptome of cucumber apical buds. A, Analysis of metabolic pathways associated with differentially expressed genes in the transcriptome. The red circle represents the plant hormone response pathway. B, Heat map of ARF and ERF transcription factor genes in DEGs. The scale from green to red indicates a gradual increase in gene expression level. C, Expression of *ARF* and *ERF* genes in the apical buds of 406a after 1 h and 2 h of 50 mg/L IAA treatments. ^*^, the result of internal reference gene *CsACTIN2*. The red dotted box indicates the *CsESR2* gene (*Csa5G98600*).

To explore the upstream factors that regulate ethylene synthesis under exogenous IAA treatment, we performed transcriptome sequencing on IAA-treated apical buds of the 406 line and identified 399 differentially expressed genes (DEGs) (Table S1). GO (Gene Ontology) term analysis showed that the DEGs were most significantly enriched in the hormone signaling response pathway (Figure 3A). We identified two auxin response factor (ARF) genes and six ethylene response factor (ERF) genes that were upregulated after IAA induction (Table S2): the *ARFs Csa2G000030* and *Csa3G866510* and the *ERFs Csa2G382550*, *Csa4G630010*, *Csa4G652640*, *Csa7G432080*, *Csa5G598600*, and *Csa1G042290*. Expression patterns of these candidate transcription factor genes are shown in a heat map (Figure 3B). The expression levels of these genes were also detected in the androecious line 406a after exogenous IAA treatment, and all were upregulated by the IAA treatment (Figure 3C). *Csa4G630010*, *Csa5G598600*, and *Csa2G000030* were particularly highly induced after 1 h of IAA treatment.

### *CsESR2* was involved in the female flower development of cucumber

**Figure 4:**
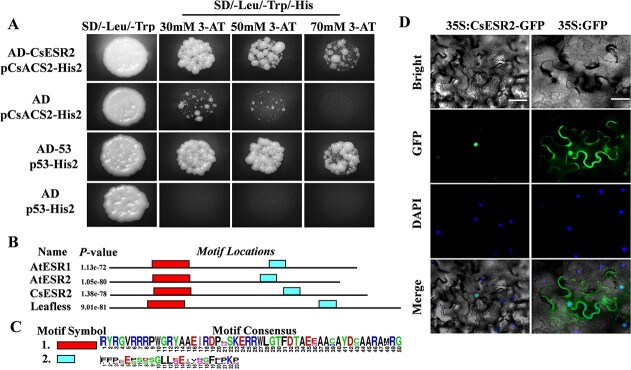
Characteristics of the candidate gene *CSESR2*. A, The results of a Y1H assay showing direct binding between CsESR2 and the *CsACS2* promoter. Vector combinations of pGADT7-53/p53-His2 and pGADT7/p53-His2 were used as the positive and negative controls, respectively. A, A series of 3-AT concentrations (30 mM, 50 mM, and 70 mM) were used for the test of yeast growth on selective medium (−Leu, −Trp, -His), and all combinations grew normally on non-selective medium (−Leu, −Trp). B, Identification of conserved motifs in the amino acid sequences of ESR homologs (AtESR1, AT1G12980; AtESR2, AT1G24590; SlESR2/LEAFLESS, SL05G013540; CsESR2, Csa5G598600). C, Conserved sequence analysis of motif1 (red rectangle) and motif2 (blue rectangle) in B. D, Subcellular localization analysis of CsESR2 in tobacco leaf epidermal cell. 35S:GFP was used as the positive control, and DAPI staining indicated the nucleus. Bar = 50 μm.

We demonstrated that sex-controlling genes (*CsACS1*, *CsACS2*, *CsACS11*, *CsACO2*, *CsWIP1*) could respond directly to induction by exogenous IAA, but whether the candidate ARF and ERF transcription factors could directly regulate these genes remained to be confirmed. A Y1H assay indicated that only Csa5G598600 showed binding activity to the promoter of *CsACS2*, whereas Csa2G000030 (ARF) and another ERF transcription factor, Csa4G630010, which were highly induced by exogenous IAA, did not bind to the promoters of these genes (Figure 4A, Figure S4). According to its gene annotation in the CuGenDB database, *Csa5G598600* encodes the ethylene response factor CsESR2 in cucumber, a homolog of *Arabidopsis* ESR2/DRNL. Sequence alignment and motif analysis revealed that the ESR2 homologs in three species (*Arabidopsis* AtESR1/AtESR2, tomato LFS, and cucumber CsESR2) were highly conserved (Figure S2), especially in the AP2 domain at the N terminus and the ESR domain at the C terminus (Figure 4B, C). Subcellular localization revealed that CsESR2 had a clear nuclear localization signal (Figure 4D) and may thus play the role of a regulator, binding to the promoters of downstream genes. *Cis-*acting elements in the 3-kb promoter sequence of *CsESR2* were predicted, and three sites of auxin response elements (−1120, −2811, −2833) were found (Table S3).

**Figure 5:**
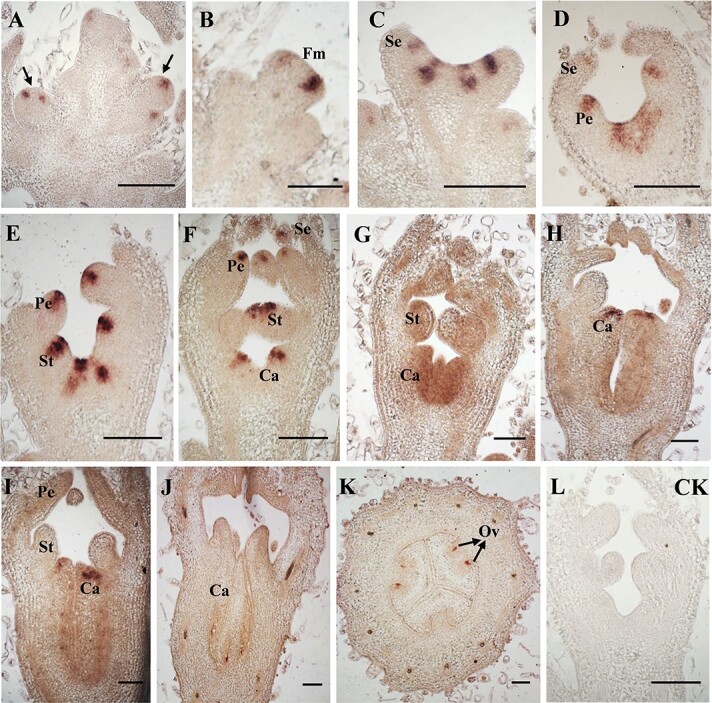
*In situ* hybridization of *CsESR2* mRNA during different developmental stages of cucumber flower buds. A, Newly initiated floral buds (early stage 1) are indicated by arrowheads. B–H, Expression patterns of CsESR2 from stage 1 to stage 7 of flower bud differentiation. I–J, Expression patterns of CsESR2 at stages 8-1 and 8-2. K, Expression pattern of *CsESR2* at stage 9. L, Negative control with no expression signal. Fm, floral meristem; Se, sepal; Pe, petal; St, stamen or stamen primordium; Ca, Carpel; Ov, ovary. Bar = 100 μm.

*In situ* hybridization was used to detect the expression pattern of *CsESR2* at different stages of floral primordium development. *CsESR2* was strongly expressed in the floral primordium at stages 1 and 2, during which stages specific floral tissues could not be identified (Figure 5A–C). At stage 3, with the differentiation of specific floral tissues, the expression of *ESR2* could be clearly identified in the petal primordia. From stage 4 to stage 5, its expression could be detected in the primordia of carpels, stamens, and petals (Figure 5D–F). Subsequently, in female flower buds, the expression of *CsESR2* was concentrated at the differentiated carpel primordium at stage 6 and was only obvious at the apex of the carpel primordium at stage 7 (Figure 5G, H). At stage 8-1, its expression was maintained at the apex of the carpel primordium and increased at the developing ovule primordium (Figure 5I). However, at stage 8-2, only the expression in the ovule area was maintained (Figure 5J). A cross-sectional view of the developing ovary showed that *CsESR2* was expressed near the site of ovule and placenta formation at stage 9 in the female flower (Figure 5K).

### CsESR2 directly activated *CsACS2* to participate in cucumber sex determination

**Figure 6:**
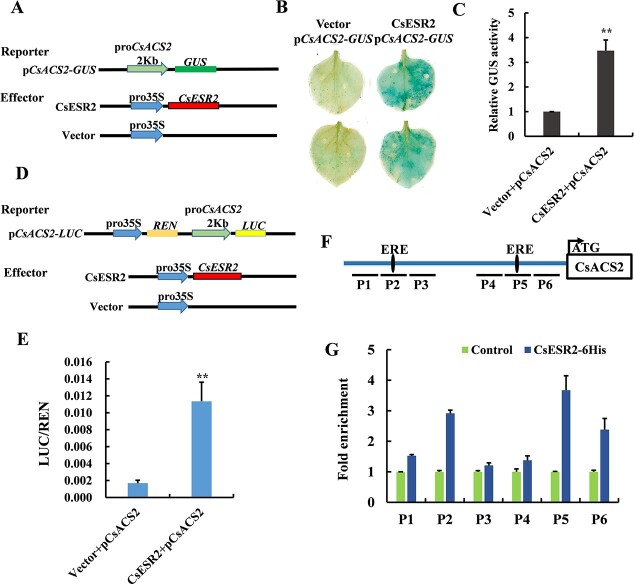
CsESR2 directly regulates the expression of *CsACS2*. A, Schematic diagram of the vectors used in the tobacco transient expression system. B, GUS staining was used to detect the effect of CsESR2 on the promoter activity of *CsACS2*. C, GUS enzyme activity assay was used to quantitatively analyze the influence of CsESR2 on the activity of the *CsACS2* promoter. The data are presented as the average of ten biological replicates, and error bars represent the SD. Student’s *t*-test was used to determine significant differences (**p<0.01). D, Schematic diagram of the vectors used in the dual luciferase reporter system. E, Luc enzyme activity assay was used to quantitatively analyze the influence of CsESR2 on the promoter activity of *CsACS2*. The data are presented as the average of ten biological replicates, and error bars represent the SD. Student’s *t*-test was used to determine significant differences (^**^p<0.01). F, Six promoter regions (P1 to P6) were used in the ChIP-PCR assay. The two black ellipses represent the two ERE elements in the promoter. G, ChIP-PCR assay in transgenic tobacco leaves with 35S:*CsESR2*-*GFP-*6His construct. Uninjected transgenic tobacco leaves were used as a negative control. An “input DNA” control was used as an internal reference for normalization. Error bars represent the SD of three biological replicates.

A tobacco transient transformation system was used to verify the regulatory relationship between *CsESR2* and *CsACS2* (Figure 6A). The effector contained the *CsESR2* CDS driven by a CaMV35S promoter. The reporter construct included the 2-kb promoter sequence of *CsACS2* (p*CsACS2*) integrated upstream of a *GUS* gene. A mixture of *Agrobacterium* solution with effector and reporter constructs was injected into tobacco leaves, and the experimental group that received CsESR2 and p*CsACS2* showed stronger catalytic activity for X-Gluc substrate than the control group (Figure 6B). Quantitative analysis showed that the GUS enzymatic activity of the experimental group was about 3.5 times higher than that of the control group (Figure 6C). A dual luciferase (LUC) reporter assay was used to further verify the activation effect, and recombinant plasmids of the corresponding reporter and effector were constructed as shown in Figure 6D. The LUC enzyme activity of the experimental group (CsESR2 and p*CsACS2*) was more than 7 times that of the control group (empty vector and p*CsACS2*) (Figure 6E). This result further confirmed that CsESR2 could upregulate the expression of *CsACS2*.

As reported previously, two EREs (ethylene-responsive elements, core sequence AWTTCAA) at −1334 bp (the distant ERE, named D-ERE) and −154 bp (the near ERE, named N-ERE) sites were predicted in the *CsACS2* promoter (Li et al., 2012). ChIP-qPCR was used to determine whether CsESR2 could bind to the EREs. Six regions of the *CsACS2* promoter, including the two ERE elements and their adjacent regions (Figure 6F), were selected for enrichment detection. The enrichment signal of CsESR2 was clearly detected in the P2 and P5 regions (Figure 6G), which contained the D-ERE and N-ERE, respectively, and the enrichment level was higher in the P5 region than in the P2 region.

## Discussion

### Exogenous auxin enhanced cucumber femaleness in a dose-dependent manner

As early as the 1960s, Galun (1962) found that auxin treatment could transform male flower buds to female flower buds in cucumber [11]. However, the suitable conditions for exogenous auxin were not clear. Our results showed that the femaleness enhancement of cucumber seedlings increased with increasing IAA concentration, which indicated that exogenous auxin may induce female flowers in a dosage-dependent manner. Higher IAA concentrations (for example, 500 mg/L in this study) can have stronger effects on femaleness enhancement, and the androecious 406an line showed the most obvious induction effect. Consistent with these results, ethylene release rates of the treated lines rose with increasing IAA concentration, indicating that femaleness enhancement after exogenous IAA treatment may depend on endogenous ethylene synthesis in cucumber.

### Cucumber sex-controlling genes can respond to regulation by exogenous auxin

After IAA treatment, ethylene biosynthesis-related genes (*CsACS1*, *CsACS2*, and *CsACS11*) that function in cucumber sex determination were upregulated, whereas the female suppressor gene *CsWIP1* was downregulated. To exclude a feedback effect of ethylene signaling on these genes [28], we used an ethylene synthesis inhibitor to first eliminate the endogenous ethylene background and then analyzed the induction effect again (Figure 2C). Among the *ACS* family members, only *CsACS1*, *CsACS2*, and *CsACS11* were directly induced by IAA treatment, and *CsACS2* showed the strongest upregulation. As a repressor of ethylene synthesis in cucumber sex determination, *CsWIP1* showed significantly lower expression under exogenous IAA treatment (Figure 2C, D), consistent with the upregulation of three *ACS* genes (Figure 2C, D) and the increase in ethylene release (Figure 2A). These results indicated that the antagonistic relationship between *CsWIP1* expression and ethylene synthesis was still present in exogenous auxin-induced cucumber sex determination. Because the mutation in *CsACO2* (in the 406a line) results in the inhibition of ethylene synthesis in sex determination [12, 15], the 406a line was used to determine the direct effect of exogenous IAA on sex-controlling genes. At 1 h of IAA treatment, *CsACS2* and *CsACS11* were upregulated, and the upregulation of *CsACS2* was greater than that of *CsACS11*. In cucumber, *CsACS2* and *CsACS11* encode ACC synthases with different functions in sex determination. The ethylene synthesized jointly by *CsACS11* and *CsACO2* can promote the expression of *CsACS2*, which then inhibits stamen development [12, 17]. Mutations in *CsACS2* and *CsACS11* lead to the emergence of andromonoecious and androecious plants, respectively [14, 29]. Such functional differences between *CsACS2* and *CsACS11* were also found in the induction of female flowers by exogenous auxin. In this study, increased ethylene release rate and femaleness were more pronounced in the 406an line (with a mutant *CsACS11* gene) than in the H38 line (with a mutant *CsACS2* gene) (Figure 2A and Table 1). Therefore, we speculated that *CsACS2* may be more sensitive than *CsACS11* in femaleness enhancement induced by exogenous auxin.

### Exogenous auxin can enhance cucumber femaleness in a *CsACO2*-independent manner

*CsACO2* can coordinate with *CsACS11* to affect female flower development through preferential ethylene production in flowers, and mutation of *CsACO2* eliminated female flowers in the 406a line [12, 15]. However, when 406a plants were treated with exogenous IAA, obvious enhancement of ethylene release and femaleness were observed, indicating that other *ACO* family gene(s) could promote ethylene synthesis. After IAA treatment, the expression of *CsACO1*, *CsACO3*, and *CsACO4* was upregulated with time, and the upregulation of *CsACO3* was the most obvious. Previous studies have shown that *CsACO1* and *CsACO3* are partly expressed in flower buds before stage 6, and the apical buds of a *CsACO2* mutant still retained some ethylene synthesis ability [15]. In this study, after exogenous IAA induction, *ACO* family members other than *CsACO2* may have also participated in sex determination-related ethylene synthesis, thus inducing female flowers.

### CsESR2 may be the link between auxin and ethylene in cucumber sex determination

It has previously been reported that crosstalk between auxin and ethylene depends on the interaction of their signaling and/or biosynthetic pathways [30]. Several *ARFs* and *ERFs* were identified in the transcriptome data (Table S2), and two *ERF* genes (*CsESR2* and *Csa4G630010*) and one *ARF* gene (*Csa2G000030*) were highly induced by auxin treatment, suggesting that they may play regulatory roles in this crosstalk (Figure 3C).

Our results revealed that ethylene synthesis-related genes (*CsACS2* and *CsACS11*) involved in sex determination responded directly to auxin induction (Figure 2C, D) and further confirmed the direct binding relationship between CsESR2 and the sex-controlling gene *CsACS2.* CsESR2 had high homology to AtESR2 in *Arabidopsis* and LFS in tomato (Figure 4B and Figure S2). *AtESR2* and *LFS* have been shown to participate in organ formation in response to auxin induction in the meristem region [31, 32]. In *Arabidopsis* pistils, an auxin marker coincided with the expression signal of *AtESR2* [33, 34]. The findings that *CsESR2* was expressed in floral organ primordia at the early growth stage and that three auxin responsive elements were present in the *CsESR2* promoter suggested that CsESR2 was likely to respond to auxin induction and participate in early floral organ development in cucumber.

**Figure 7:**
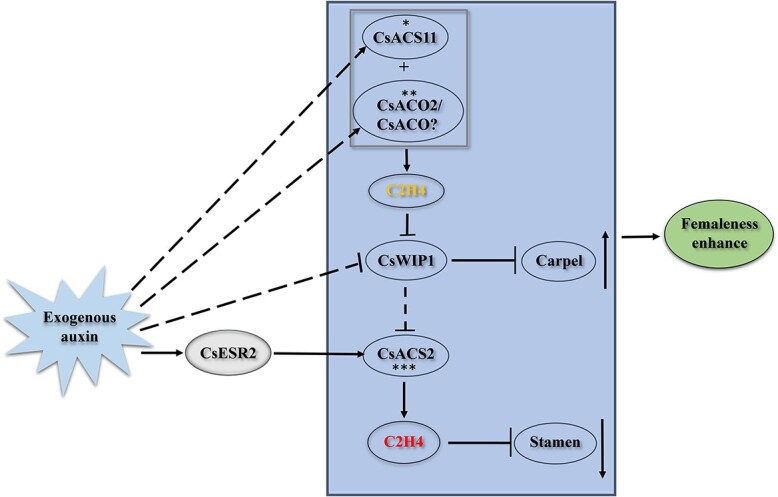
A mechanistic model of the enhancement of femaleness in materials of different sex types in response to exogenous auxin treatment. The square box contains sex-controlling genes. ➜, positive regulation; $\dashv$, negative regulation; +, synergy; **—**, direct effect; ­­­ indirect effect. ^*^, *CsACS11* mutation in 406an; ^**^, *CsACO2* mutation in 406a; ^***^, *CsACS2* mutation in H38; *CsACO*?, *CsACO* genes induced by exogenous auxin, excluding *CsACO2*; yellow C2H4, ethylene in the early stage; red C2H4, ethylene in the late stage.

### CsESR2 is involved in enhancing femaleness through activation of *CsACS2*

Previous studies have confirmed that *AtESR2* is expressed in the founder cells of lateral organs and floral organs [33]. *AtESR2* is also expressed in the ovary region during late pistil development, and its mutation caused pistil dysplasia [35]. We demonstrated that *CsESR2* was expressed at different stages and positions in the developing cucumber flower (Figure 5). Previous studies have shown that stage 4 is the key period for the sex determination of a flower bud, with the co-occurrence of *CsACO2* and *CsACS11* at stage 4 in female flower buds [15]. The expression of *CsACS2* begins from the initiation of the carpel primordium at stage 5 and continues until ovary formation [37]. *CsESR2* is strongly expressed at the early stage of the flower primordium, and at stage 5, its expression region overlaps with that of *CsACS2* at the upper part of the carpel primordium [37]. From stage 5 to stage 8, the expression regions of *CsESR2* and *CsACS2* continue to overlap and shift from the upper to the lower part of the carpel primordium. In addition, both are expressed near the site of ovule and placenta formation in stage 9 of the ovary based on cross sectional analysis [37]. The earlier transcription of *CsESR2* and its similar expression pattern to *CsACS2* after stage 5 support the possibility that CsESR2 may regulate the expression of *CsACS2* during flower development.

The direct activation of *CsACS2* by CsESR2 was demonstrated in this study (Figure 4A and Figure 6). Previous studies have reported that CsERF31 binds to the D-ERE and triggers a positive feedback loop of ethylene during female flower differentiation [38]. Similarly, our ChIP-qPCR result showed that CsESR2 can bind to the promoter of *CsACS2* through the ERE element regions (Figure 6F, G). This result demonstrated that CsESR2, as an upstream regulator of *CsACS2*, could activate its expression.

In Figure 7, we summarize the femaleness enhancement mechanisms of several materials with different sex types under exogenous auxin treatment. For monoecious material (406) with no mutations in sex-controlling genes, exogenous auxin regulates the expression of these genes to promote normal ethylene synthesis and increase the number of female flowers. For androecious materials (^*^, the *CsACS11* mutation in the 406an mutant; ^**^, the *CsACO2* mutation in the 406a mutant), mutation of *CsACS11* or *CsACO2* leads to early obstruction of ethylene synthesis during sex determination. This releases *CsWIP1* and inhibits *CsACS2* expression [12, 15], resulting in the phenotype of all-male flowers. However, exogenous auxin can inhibit *CsWIP1* expression and directly promote *CsACS2* expression through *CsESR2*, thus bypassing the dependence on early ethylene synthesis and inducing female flowers. For andromonoecious materials (^***^, the *CsACS2* mutation in the H38 mutant), early ethylene can promote carpel development, but late ethylene that relies on *CsACS2* cannot be synthesized normally, and stamen development cannot be inhibited, resulting in bisexual flowers [12]. Under exogenous auxin treatment, the expression of *CsACS11* and *CsACO2* is activated, and increased ethylene synthesis in the early stage promotes carpel development; however, stamens are still not inhibited owing to the absence of late ethylene, resulting in an increased number of bisexual flowers under exogenous auxin treatment.

In the 406a mutant, the number of female flowers increased after treatment with exogenous auxin, indicating that *CsACO* genes (*CsACO?*) other than *CsACO2* may be involved in cucumber sex determination under exogenous auxin treatment. However, further experiments need to be performed to identify possible candidate *CsACO* genes. In conclusion, we demonstrated that CsESR2 acts as a bridge between exogenous auxin signaling and endogenous ethylene synthesis in cucumber sex determination and constructs a crosslink between the two hormones.

## Materials and methods

### Materials and growth conditions

The cucumber inbred lines used in the following experiments were 406 [15] (monoecious line, *ffMMAA*), GY14M-17 [39] (androecious line, *ffMMAA*), H38 [13] (*CsACS2* mutant, andromonoecious line, *ffmmAA*), 406a [15] (*CsACO2* mutant, androecious line, *ffMMAA*&*aco2aco2*) and 406an (*CsACS11* mutant, androecious line, *ffMMaa*). The 406a and 406an mutants were identified from an EMS mutagenized mutant library of the 406 line. In 406an, a change from C to T at the 902th nucleotide of the CsACS11 CDS results in an amino acid change from tryptophan (S) to phenylalanine (F) in the conserved BOX6 motif (Figure S3). This amino acid has been shown to be highly conserved in a variety of plants [14]. Cucumber seedlings at the two-leaf stage were grown in light incubators at 28°C/16 h light and 20°C/8 h darkness. Cucumber seedlings with two fully expanded true leaves were transferred to the horticultural greenhouses at Northwest A&F University in Yangling, China.

### Exogenous auxin treatment

IAA (Sigma, USA) solutions of four concentrations (0, 10, 50, and 500 mg/L) were prepared. The apical buds of cucumber seedlings at the two-leaf stage were sprayed with IAA solution, and this was repeated every 5 days for a total of four applications. For each cucumber line with a different sex type, 15 seedlings were treated with each concentration of IAA solution. This experiment was repeated in three cucumber growing seasons.

### Number of female nodes in cucumber

A node with one or more blooming female flowers is considered to be a female node. The numbers of female flower nodes of 7-week-old plants treated with IAA solution were counted for the first 20 nodes (bisexual flower nodes in H38 were recorded). Fifteen plants were counted in each treatment, and the experiment was repeated in three cucumber growing seasons.

### Determination of ethylene release rate

About 0.5 g of apical buds were excised from plants treated with IAA solution and placed in a 12-mL container sealed with a rubber stopper. After incubation at 28°C for 16 h, 1 mL headspace gas was withdrawn using a gas-tight syringe. The gas was analyzed using a TRACE GC Ultra gas chromatograph (Thermo Scientific, USA), and the instrument was calibrated with an ethylene gas standard before measurement. The ethylene release rate (μL/kg·h) of cucumber apical buds was calculated from the measured data. Three biological replicates were performed for each IAA-treated line.

### Gene expression analysis

When 406 and 406a plants had grown to the four-leaf stage, they were treated with 50 mg/L IAA. The apical buds of 406 were collected for RNA extraction after one hour, and 406a buds were collected after one and two hours. Meanwhile, naturally growing 406 plants were treated with 1 mM AVG (Sigma, USA) in H_2_O containing 0.1% (V/V) Tween20 using a pipettor once a day for 3 days, and then 50 mg/L IAA was sprayed on the plants. Solution without IAA was used as the control. RNA extraction and first-strand cDNA synthesis were carried out as described previously [28]. Q311-02 SYBR qPCR Master Mix (Vazyme, China) and an ABI 7500 instrument (Thermo Scientific, USA) were used for quantitative real-time PCR (qRT-PCR). Three replicates were measured for each sample, and *CsACTIN2* was used as the reference gene. Relative gene expression was calculated using the 2^−ΔΔCt^ formula. Semi-quantitative PCR was performed for 33 cycles (94°C, 20 s; 60°C, 20 s; 72°C, 20 s), and the products were detected on a 1.5% agarose gel. *CsACTIN2* was used as the reference gene to balance the added volume of different cDNA samples. The related primers are listed in Supplemental Table S4.

### Transcriptome analysis

Apical buds of the 406 line at the four-leaf stage were sprayed with 50 mg/L IAA, and the tissues were harvested after 1 h. Control buds were treated with solution without IAA. At least six apical buds were mixed into one sample, and three sample replicates were analyzed by RNA-seq. RNA-seq libraries were constructed using the NEBNext Ultra Directional RNA Library Prep Kit (NEB, USA). DEGs were required to show at least a 1.5-fold expression change between the IAA-treated plants and controls with a false discovery rate (FDR) less than 0.05.

### Cloning of gene sequences and subcellular localization analysis

The cDNA and genomic DNA of the 406 line were used to clone coding sequences (CDSs) and promoter sequences of the target genes, respectively. Genomic DNA of the 406 line was extracted as described previously [29]. All sequences were amplified using high-fidelity DNA polymerase (Vazyme, China) with specific primers (Supplemental Table S4). The PCR product was fused with the pMD19-T cloning vector (Takara, Japan) and identified by sequencing.

The *CsESR2* cDNA was fused to the pGreenII-GFP vector for detection of subcellular localization, and all operations were performed as described previously [40]. DAPI was used to stain and label the nucleus. Specific gene primers are listed in Supplemental Table S4.

### Bioinformatics analysis

Cucumber gene information in this study was obtained from the Cucurbitaceae Genome Database (CuGenDB, http://cucurbitgenomics.org/). The *AtESR2* sequence of *A. thaliana* was obtained from the TAIR database (https://www.arabidopsis.org/), and the *LFS* (*SlESR2*) sequence of *Solanum lycopersicum* was obtained from the Solanaceae Genome Database (https://solgenomics.net/). Amino acid sequences of ESR2 homologs in cucumber, tomato and *Arabidopsis* were aligned using ClustalW2 (http://www.ebi.ac.uk/Tools/msa/clustalw2/). Conserved motifs of ESR2 homologs were analyzed using the MEME program (http://meme-suite.org/tools/meme). Putative *cis*-acting elements in the *CsACS2* promoter were identified using the PlantCARE database [41].

### Yeast one-hybrid (Y1H) assay

Promoter sequences (2 kb) of *CsACS1*, *CsACS2*, *CsACS11*, *CsACO2*, and *CsWIP1* were ligated into the pHis2 bait vector, and the CDSs of *CsESR2*, *Csa4G630010*, and *Csa2G000030* were cloned into the pGADT7 prey vector. The yeast strain Y187 was used for transformation with a yeast transformation kit (Coolaber, China). Three concentrations (30, 50, and 70 mM) of 3-amino-1,2,4-triazole (3-AT) were used to inhibit background activity of the recombinant bait vector. The combination of pGADT7-53 and p53-His2 was transformed as a positive control, and the combination of pGADT7 and p53-His2 was transformed as a negative control. Specific primers are listed in Supplemental Table S4.

### *In situ* hybridization assay

Cucumber flower buds of different developmental stages were fixed in 3.7% formalin-acetic acid-alcohol (FAA) solution and used for *in situ* hybridization (ISH) as described previously [12]. Sense and antisense probes were synthesized using SP6 and T7 polymerase, respectively. Probes for *CsESR2* were designed using the 5′ region of the CDS sequence. Probe primers are listed in Supplemental Table S4. The stages of cucumber flower bud differentiation were divided according to Bai et al. (2004) [36].

### Transient expression assay in tobacco leaves and GUS staining

The *CsESR2* cDNA was fused into the pCAMBIA1305.4 vector to construct the pCAMBIA1305.4-35S:CsESR2-6xHis effector, and the pBI121-proCsACS2:GUS plasmid was used as a reporter [42]. All the vectors were transformed into the GV3101 (pSoup) *Agrobacterium* strain. A mixed bacterial solution of effector and reporter was injected into tobacco (*N. benthamiana*) leaves in a volume ratio of 9 to 1, and a bacterial mixture of the pCAMBIA1305.4 empty vector and the effector was injected as the negative control. Measurement of GUS activity was performed as described previously [43]. Each bacterial solution combination was injected independently into ten tobacco leaves. Related primers are listed in Supplemental Table S4.

### Dual luciferase reporter analysis

The *CsESR2* cDNA was fused into the pGreenII62-SK vector to construct the pGreenII62-SK-35S:CsESR2 effector, and the pGreenII0800-p*CsACS2*:LUC plasmid was used as the reporter [42]. A mixed bacterial solution of effector and reporter was injected into tobacco leaves in a volume ratio of 9 to 1, and the bacterial mixture of the pGreenII0800 empty vector and the effector was injected as the negative control. Each bacterial solution combination was injected independently into ten tobacco leaves. After 3 d, the injected leaves were removed and ground with liquid nitrogen. The samples were processed according to the instructions of the Dual-Luciferase Reporter Assay System kit (Promega, USA). The luciferase activities of firefly luciferase (LUC) and renilla luciferase (REN) in the samples were detected using an Infinite M200 PRO full-wavelength microplate reader (Tecan, Switzerland). Related primers are listed in Supplemental Table S4.

### ChIP-qPCR (chromatin immunoprecipitation assay-qPCR) assay

Transgenic tobacco leaves containing the *CsACS2* promoter-GUS fusion (transformed with the pBI121-proCsACS2:GUS vector [42]) were infiltrated with GV3101 *Agrobacterium* solution containing the pCAMBIA1305.4-35S:CsESR2-6His fusion vector (effector) or the pCAMBIA1305.4 empty vector (negative control). Positive infiltration was confirmed by observation of the GFP signal using a BX63 fluorescence microscope (Olympus, Japan). The ChIP assay was performed as described by Chen et al. (2016) [15] using an anti-6xHis antibody (Thermo Fisher Scientific, USA). After the ultrasonic treatment, 20-μL DNA samples from infiltrated leaves containing the effector or the negative control were extracted separately for “input DNA” control. The binding intensity between CsESR2 and the *CsACS2* promoter fragment was examined by qPCR in triplicate, and the “input DNA” control was used to normalize the results. One microliter of the immunoprecipitated chromatin product was used as the qPCR template. The enrichment level was assessed in six regions of the *CsACS2* promoter. Related primers are listed in Supplemental Table S4.

## Supplementary Material

Web_Material_uhab085

## Data Availability

The data that support the results are included in this article and its supplementary materials. Other relevant materials are available from the corresponding author upon reasonable request.
