# Supplementary material for: Exogenous auxin-induced ENHANCER OF SHOOT REGENERATION 2 (ESR2) enhances femaleness of cucumber by activating the CsACS2 gene
Source: Hortic Res. 2022 Jan 20;9:uhab085. doi: 10.1093/hr/uhab085 (PMC9039497; doi:10.1093/hr/uhab085)
Supplement: Web_Material_uhab085 [file web_material_uhab085.zip › Supplemental Figures.pdf]

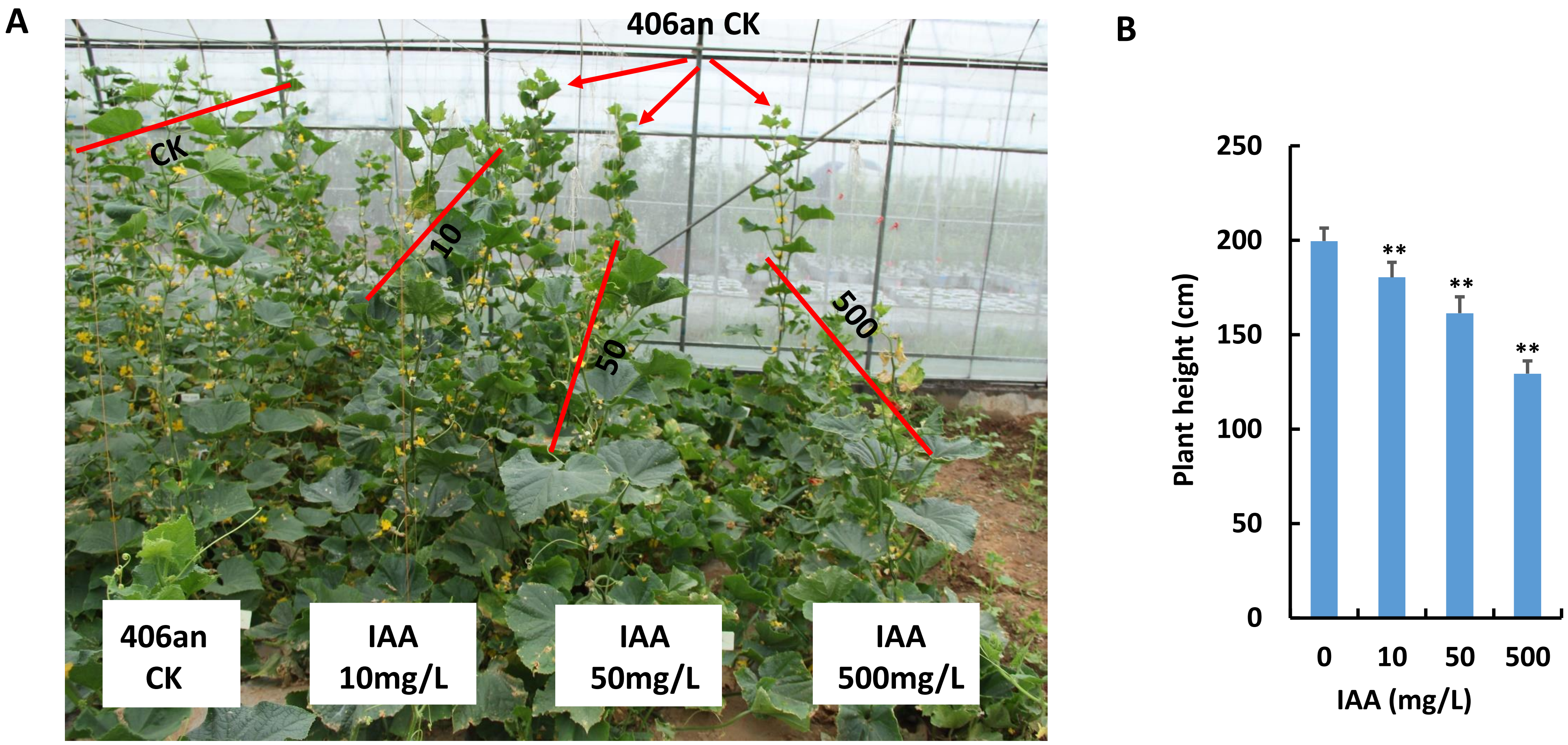
A
406an CK
CK
10
50
500
406an
CK
IAA
10mg/L
IAA
50mg/L
IAA
500mg/L
B
250
200
150
100
50
0
Plant height (cm)
**
**
**
0
10
50
500
IAA (mg/L)

Figure S1.

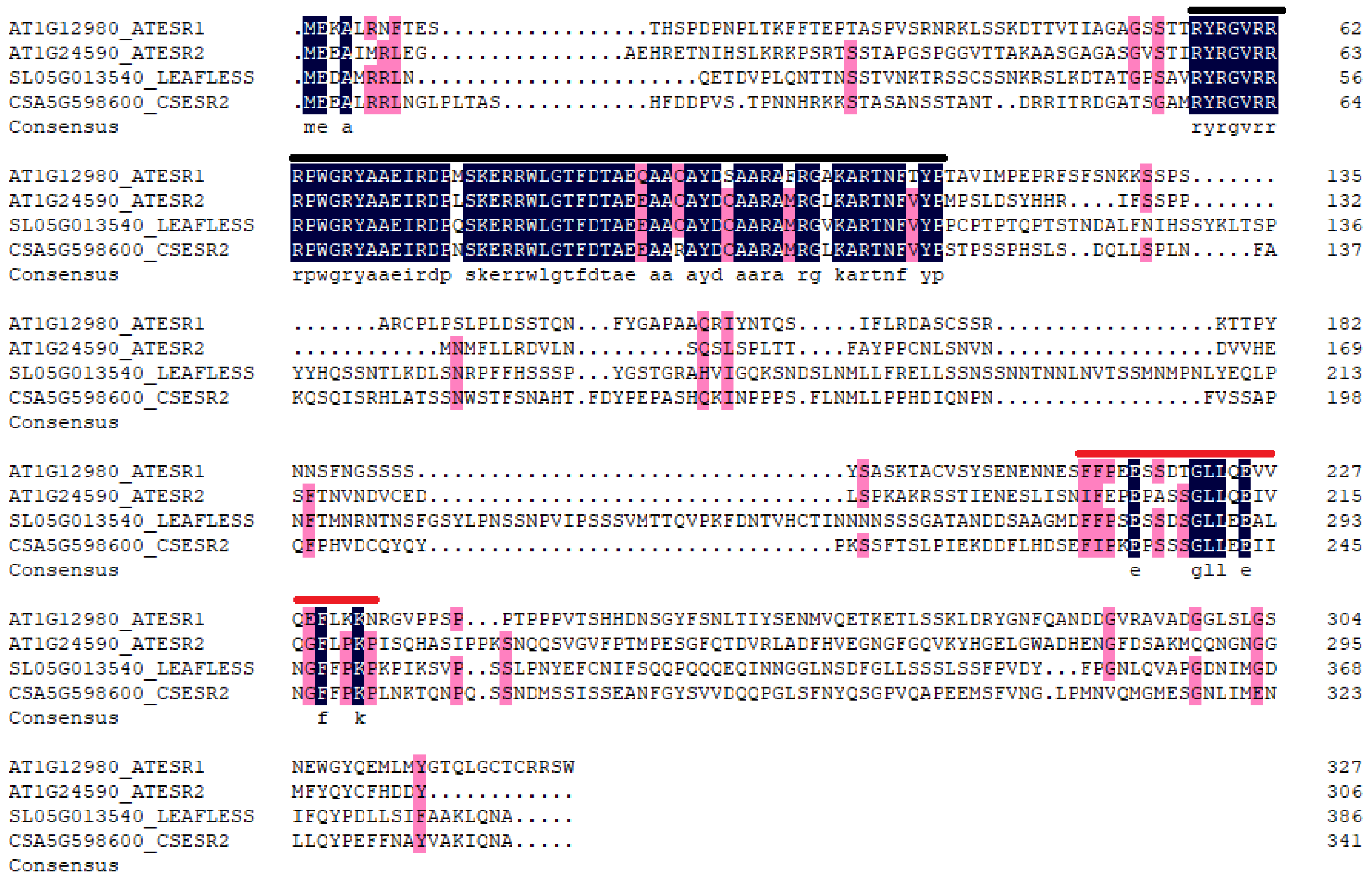
AT1G12980_ATESR1 .MEKALRNFTES.................THSPDPNPLTKFFTEPTASPVSRNRKLSSKDTTVTIAGAGSSTTRYRGVRR 62
AT1G24590_ATESR2 .MEEAIMRLEG................AEHRETNIHSLKRKPSRTSSTAPGSPGGVTTAKAASGAGASGVSTIRYRGVRR 63
SL05G013540_LEAFLESS .MEDAMRRLN.......................QETDVPLQNTTNSSTVNKTRSSCSSNKRSLKDTATGPSAVRYRGVRR 56
CSA5G598600_CSESR2 .MEEALRRLNGLPLTAS............HFDDPVS.TPNNHRKKSTASANSSTANT..DRRITRDGATSGAMRYRGVRR 64
Consensus me a ryrgvrr
AT1G12980_ATESR1 RPWGRYAAEIRDPMSKERRWLGTFDTAEQAACAYDSAARAFRGAKARTNFTYPTAVIMPEPRFSFSNKKSSPS....... 135
AT1G24590_ATESR2 RPWGRYAAEIRDPLSKERRWLGTFDTAEEAACAYDCAARAMRGLKARTNFVYPMPSLDSYHHR....IFSSPP....... 132
SL05G013540_LEAFLESS RPWGRYAAEIRDPQSKERRWLGTFDTAEEAACAYDCAARAMRGVKARTNFVYPPCPTPTQPTSTNDALFNIHSSYKLTSP 136
CSA5G598600_CSESR2 RPWGRYAAEIRDPNSKERRWLGTFDTAEEAARAYDCAARAMRGLKARTNFVYPSTPSSPHSLS..DQLLSPLN.....FA 137
Consensus rpwgryaaeirdp skerrwlgtfdtae aa ayd aara rg kartnf yp
AT1G12980_ATESR1 .......ARCPLPSLPLDSSTQN...FYGAPAAQRIYNTQS.....IFLRDASCSSR..................KTTPY 182
AT1G24590_ATESR2 ............MNMFLLRDVLN.........SQSLSPLTT....FAYPPCNLSNVN..................DVVHE 169
SL05G013540_LEAFLESS YYHQSSNTLKDLSNRPFFHSSSP...YGSTGRAHVIGQKSNDSLNMLLFRELLSSNSSNNTNNLNVTSSMNMPNLYEQLP 213
CSA5G598600_CSESR2 KQSQISRHLATSSNWSTFSNAHT.FDYPEPASHQKINPPPS.FLNMLLPPHDIQNPN.................FVSSAP 198
Consensus
AT1G12980_ATESR1 NNSFNGSSSS...................................YSASKTACVSYSENENNESFFPEESSDTGLLQEVV 227
AT1G24590_ATESR2 SFTNVNDVCED..................................LSPKAKRSSTIENESLISNIFEPEPASSGLLQEIV 215
SL05G013540_LEAFLESS NFTMNRNTNSFGSYLPNSSNPVIPSSSVMTTQVPKFDNTVHCTINNNNSSSGATANDDSAAGMDFFPSESSDSGLLEEAL 293
CSA5G598600_CSESR2 QFPHVDCQYQY.................................PKSSFTSLPIEKDDFLHDSEFIPKEPSSSGLLEEII 245
Consensus e gll e
AT1G12980_ATESR1 QEFLKKNRGVPPSP...PTPPPVTSHHDNSGYFSNLTIYSENMVQETKETLSSKLDRYGNFQANDDGVRAVADGGLSLGS 304
AT1G24590_ATESR2 QGFLPKPISQHASIPPKSNQQSVGVFPTMPESGFQTDVRLADFHVEGNGFGQVKYHGELGWADHENGFDSAKMQQNGNGG 295
SL05G013540_LEAFLESS NGFFPKPKPIKSVP..SSLPNYEFCNIFSQQPQQQEQINNGGLNSDFGLLSSSLSSFPVDY...FPGNLQVAPGDNIMGD 368
CSA5G598600_CSESR2 NGFFPKPLNKTQNPQ.SSNDMSSISSEANFGYSVVDQQPGLSFNYQSGPVQAPEEMSFVNG.LPMNVQMGMESGNLIMEN 323
Consensus f k
AT1G12980_ATESR1 NEWGYQEMLMYGTQLGCTCRRSW 327
AT1G24590_ATESR2 MFYQYCFHDDY............ 306
SL05G013540_LEAFLESS IFQYPDLLSIFAAKLQNA..... 386
CSA5G598600_CSESR2 LLQYPEFFNAYVAKIQNA..... 341
Consensus

Figure S2.

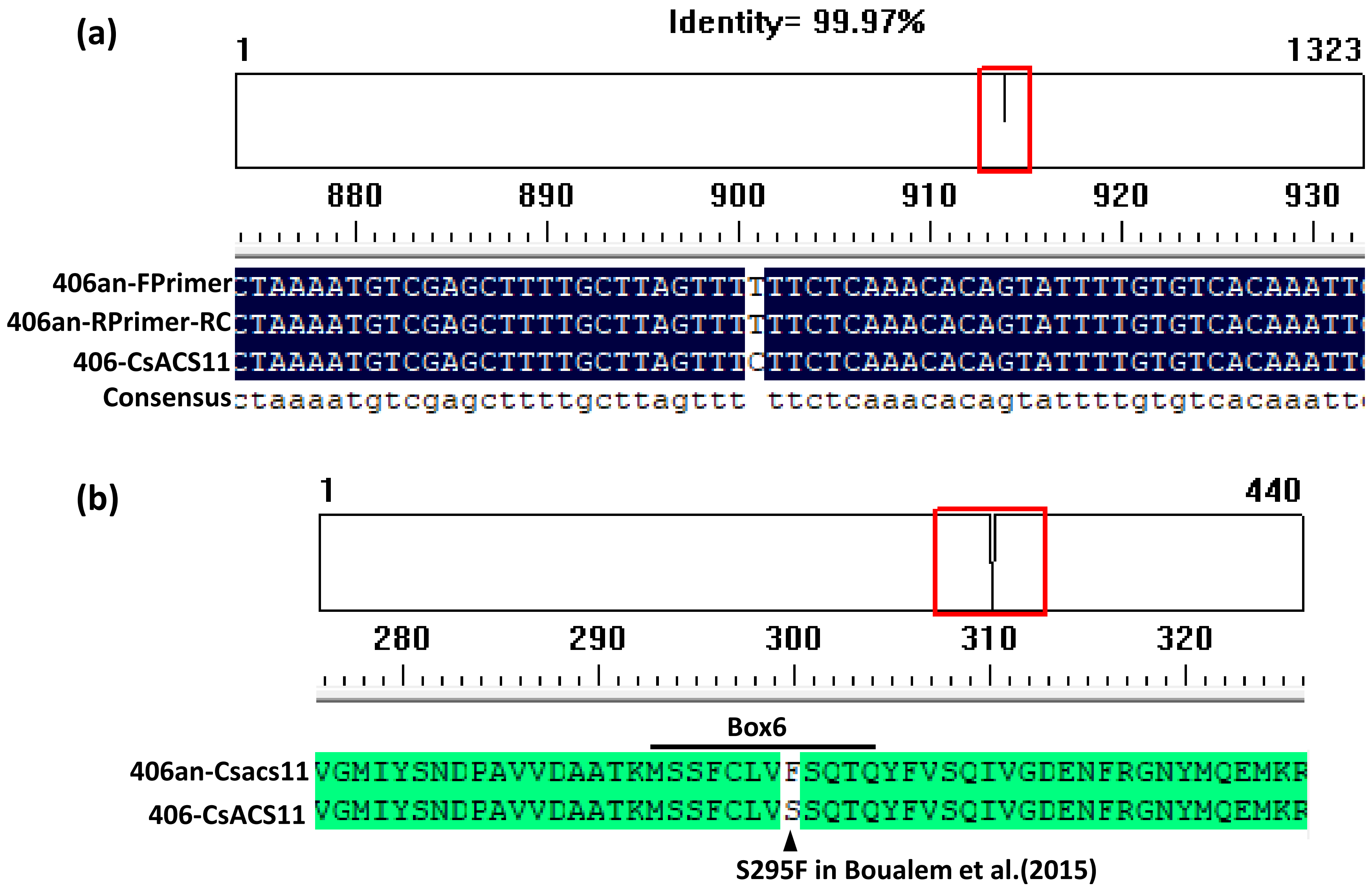
(a)
Identity= 99.97%
1
1323
880
890
900
910
920
930
406an-FPrimer CTAAAATGTCGAGCTTTTGCTTAGTTTTTTCTCAAACACAGTATTTTGTGTCACAAATT
406an-RPrimer-RC CTAAAATGTCGAGCTTTTGCTTAGTTTTTTCTCAAACACAGTATTTTGTGTCACAAATT
406-CsACS11 CTAAAATGTCGAGCTTTTGCTTAGTTTCTTCTCAAACACAGTATTTTGTGTCACAAATT
Consensus ctaaaatgtcgagcttttgcttagttt ttctcaaacacagtattttgtgtcacaaatt
(b)
1
440
280
290
300
310
320
Box6
406an-Csacs11 VGMIYSNDPAVVDAATKMSSFCLVFSQTQYFVSQIVGDENFRGNYMQEMKR
406-CsACS11 VGMIYSNDPAVVDAATKMSSFCLVSSQTQYFVSQIVGDENFRGNYMQEMKR
S295F in Boualem et al.(2015)

Figure S3.

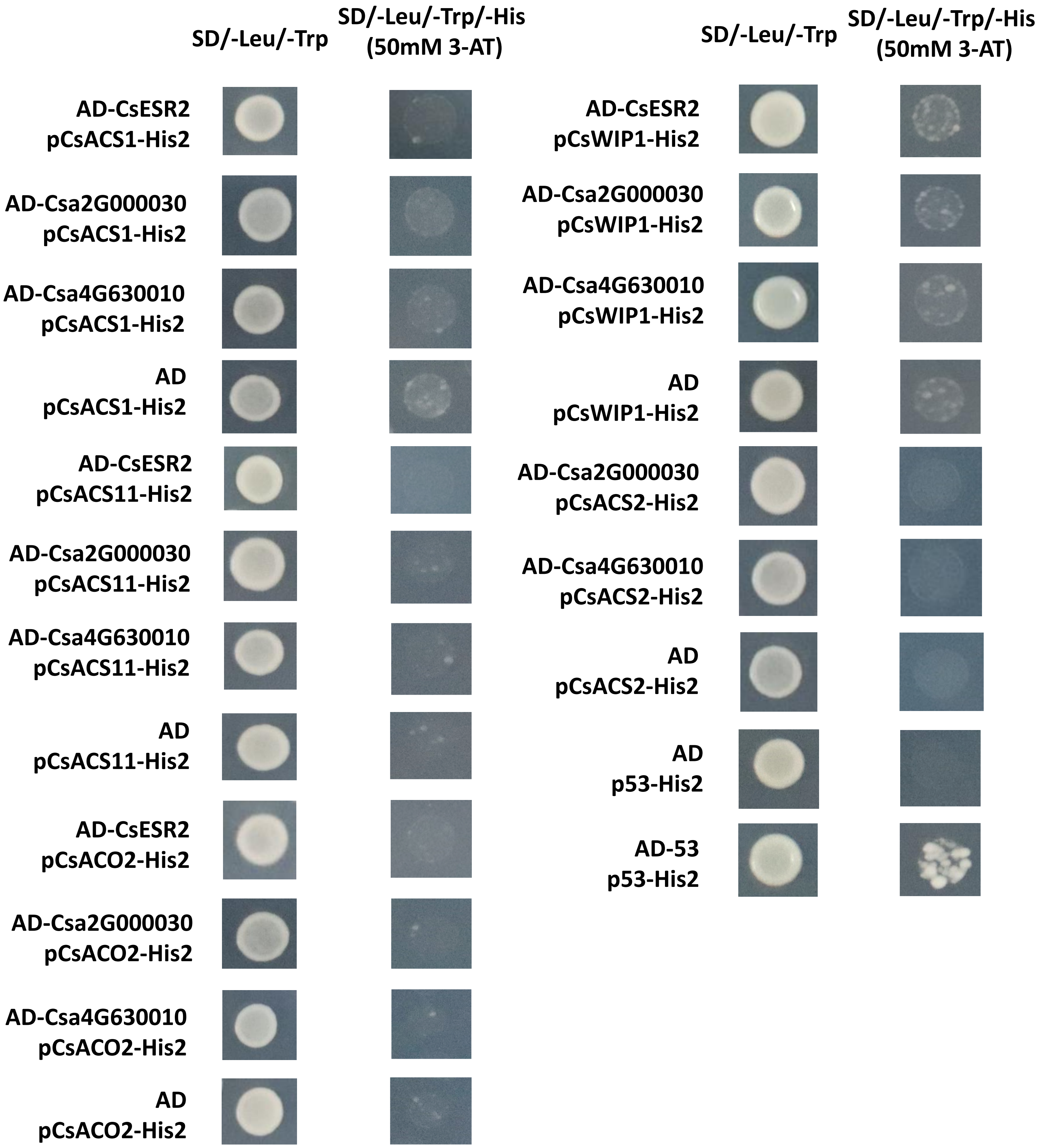
SD/-Leu/-Trp
SD/-Leu/-Trp/-His (50mM 3-AT)
AD-CsESR2 pCsACS1-His2
AD-Csa2G000030 pCsACS1-His2
AD-Csa4G630010 pCsACS1-His2
AD pCsACS1-His2
AD-CsESR2 pCsACS11-His2
AD-Csa2G000030 pCsACS11-His2
AD-Csa4G630010 pCsACS11-His2
AD pCsACS11-His2
AD-CsESR2 pCsACO2-His2
AD-Csa2G000030 pCsACO2-His2
AD-Csa4G630010 pCsACO2-His2
AD pCsACO2-His2
SD/-Leu/-Trp
SD/-Leu/-Trp/-His (50mM 3-AT)
AD-CsESR2 pCsWIP1-His2
AD-Csa2G000030 pCsWIP1-His2
AD-Csa4G630010 pCsWIP1-His2
AD pCsWIP1-His2
AD-Csa2G000030 pCsACS2-His2
AD-Csa4G630010 pCsACS2-His2
AD pCsACS2-His2
AD p53-His2
AD-53 p53-His2

Figure S4.
