# Supplementary material for: Exogenous auxin-induced ENHANCER OF SHOOT REGENERATION 2 (ESR2) enhances femaleness of cucumber by activating the CsACS2 gene
Source: Hortic Res. 2022 Jan 20;9:uhab085. doi: 10.1093/hr/uhab085 (PMC9039497; doi:10.1093/hr/uhab085)
Supplement: Web_Material_uhab085 [file web_material_uhab085.zip › Supplemental Tables.pdf]

Supplemental Table S1. RNA-Seq analysis of differentially expressed genes between auxin-treated 406 plants and controls

Symbol	IAA1	IAA2	IAA3	CK1	CK2	CK3	Case_Mean	Control_Mean	baseMean	log2FoldChange	lfcSE
Csa1G231530	7238.566	6309.593	6412.915	552.8141	590.0343	410.6296	6653.691395	517.8259945	3585.7587	3.681324866	0.106645
Csa6G493310	21089.87	25997.87	31836.16	1371.292	1675.587	1133.91	26307.96514	1393.596302	13850.7807	4.238030021	0.11897
Csa3G198490	18909.47	19064.96	24078.9	2602.915	2863.914	2129.109	20684.44428	2531.979287	11608.2118	3.02985736	0.107307
Csa3G363150	2589.56	2416.774	2755.486	191.4339	321.1695	285.1288	2587.273285	265.9107418	1426.59201	3.28219759	0.117685
Csa1G397130	5895.403	3958.816	4428.46	648.5311	519.377	572.4595	4760.893034	580.1225039	2670.50777	3.036949989	0.115005
Csa5G608220	1110.713	1472.902	1305.515	221.7117	218.3953	226.782	1296.376655	222.29632	759.336488	2.544450923	0.109248
Csa6G067980	916.1278	790.9252	938.9598	144.5521	97.26848	110.0884	882.0042693	117.3029816	499.653625	2.91137486	0.118688
Csa6G492310	1226.412	1460.331	1523.101	139.6686	172.5139	88.07069	1403.281371	133.4177281	768.34955	3.387968225	0.125887
Csa7G081680	1658.707	1680.323	1979.941	463.9341	454.2254	508.6082	1772.990378	475.5892623	1124.28982	1.899934811	0.098808
Csa5G633200	873.0035	953.3006	975.9765	167.993	146.8203	215.7732	934.0935325	176.8621721	555.477852	2.406338315	0.115135
Csa6G092540	1418.894	2013.455	1556.507	309.615	350.5336	285.1288	1662.951587	315.0924702	989.022029	2.398218719	0.115305
Csa6G507490	482.7815	355.1307	575.1129	10.74374	30.2817	20.91679	471.0083358	20.64740736	245.827872	4.505099936	0.133624
Csa6G490030	1917.452	2476.486	2253.504	605.5561	521.2122	484.3888	2215.814068	537.0523766	1376.43322	2.044075026	0.106496
Csa7G378530	923.4904	751.117	789.9902	171.8998	178.0197	136.5096	821.5325807	162.1430124	491.837797	2.33706344	0.115154
Csa3G877650	749.9416	1025.584	939.8627	187.5271	189.9488	180.5449	905.1293498	186.0069293	545.56814	2.282338367	0.114864
Csa3G002440	2252.98	2088.881	2482.826	510.8159	669.8678	503.1038	2274.895736	561.2625062	1418.07912	2.01778056	0.107629
Csa1G042820	7269.069	7900.871	8781.983	2824.626	2965.771	2658.634	7983.974425	2816.343717	5400.15907	1.503176944	0.08841
Csa1G042090	1600.857	1690.799	1565.535	617.2766	520.2946	557.0471	1619.063673	564.872753	1091.96821	1.519269239	0.093688
Csa3G150200	484.8851	438.9373	510.108	103.5306	68.82204	93.5751	477.9767874	88.64256824	283.309678	2.435174512	0.123762
Csa6G495070	10477.09	10911.63	13185.16	4613.947	4222.92	4307.757	11524.62727	4381.541548	7953.08441	1.395315944	0.093081
Csa5G598100	3090.222	3622.542	2679.647	1044.096	991.0373	1072.261	3130.80371	1035.797969	2083.30084	1.595774594	0.102099
Csa3G733950	2148.851	2122.403	2079.254	910.2876	1000.214	929.1457	2116.836124	946.5489812	1531.69255	1.160621219	0.083046
Csa3G143580	1822.789	1547.28	1508.656	420.9592	503.7773	325.8615	1626.241686	416.8660104	1021.55385	1.961288819	0.116782
Csa3G176350	292.4036	238.8489	286.2022	44.92836	45.88136	37.43004	272.4848993	42.74658588	157.615743	2.667965101	0.129695
Csa6G524040	423.88	534.2674	494.7596	114.2743	128.4678	124.3998	484.3023248	122.3806494	303.341487	1.98456834	0.118677
Csa1G004220	216.6732	257.7054	211.266	21.48748	14.68203	33.02651	228.5481837	23.06533906	125.806761	3.328223027	0.133671
Csa5G435070	1070.744	1266.528	952.5025	343.7996	244.0888	243.2953	1096.591495	277.0612346	686.826365	1.983694152	0.120291

Csa2G348990	1071.796	1031.869	1204.397	442.4467	442.2963	450.2614	1102.687242	445.0014431	773.844343	1.309993735	0.097084
Csa3G019370	3304.792	3583.782	4017.665	1413.29	1614.106	1620.501	3635.412621	1549.298889	2592.35576	1.230856025	0.094118
Csa3G143570	1313.712	1126.152	1183.631	343.7996	332.181	473.3799	1207.831792	383.1201922	795.475992	1.659122319	0.114587
Csa2G170820	1079.159	1201.578	1118.626	522.5363	462.4841	479.9852	1133.12083	488.3352216	810.728026	1.214387608	0.095662
Csa6G495080	2217.219	2167.449	2866.536	926.8916	847.8875	973.1811	2417.067966	915.9867115	1666.52734	1.40080514	0.105839
Csa5G608330	733.1126	614.9313	885.6919	219.7583	245.0065	213.5714	744.5785835	226.1120461	485.345315	1.719184024	0.118816
Csa3G728030	676.3148	771.0211	854.9952	291.0576	286.2997	264.2121	767.4436811	280.5231186	523.9834	1.451829809	0.109024
Csa3G134550	1500.935	1784.034	1542.964	764.7588	705.6553	683.6487	1609.310892	718.0209253	1163.66591	1.163820824	0.096277
Csa2G317400	558.5119	467.222	629.2837	183.6202	160.5848	179.444	551.6725258	174.5496738	363.1111	1.662276994	0.118553
Csa1G060770	4207.246	4234.331	4160.314	1863.55	1445.263	2000.305	4200.630273	1769.706127	2985.1682	1.247734668	0.102024
Csa2G200440	5585.119	4573.748	4805.849	2119.446	2406.018	1851.686	4988.238535	2125.717015	3556.97778	1.230045496	0.101753
Csa3G177390	1471.484	980.5377	2097.311	245.1526	367.9685	273.0191	1516.444399	295.3800603	905.91223	2.359554132	0.132491
Csa5G606770	1533.541	1574.517	1539.352	738.3878	800.1709	823.4609	1549.136945	787.3398647	1168.23841	0.97675373	0.086652
Csa2G379350	137.7873	170.756	119.1757	507.8858	548.741	422.7393	142.5730026	493.1220406	317.847522	-1.794433413	0.12386
Csa4G430820	6264.589	6661.581	7898.097	3094.196	3499.83	3026.329	6941.422122	3206.785141	5074.10363	1.114058899	0.096399
Csa4G377750	452.2789	467.222	391.8352	136.7385	126.6325	165.1325	437.1120413	142.8345216	289.973282	1.616560331	0.120068
Csa3G914050	337.6315	329.9887	390.9323	119.1578	108.28	115.5928	352.8508171	114.3435331	233.597175	1.62769294	0.120565
Csa1G690240	1103.35	932.3489	1052.718	331.1025	299.1465	456.8667	1029.472517	362.3718668	695.922192	1.509251155	0.118785
Csa1G532200	646.864	629.5974	835.1325	225.6185	277.1234	267.5147	703.8646664	256.7522015	480.308434	1.456040631	0.117621
Csa3G866510	13443.2	18079.19	17236.23	7624.147	7883.335	7929.664	16252.87443	7812.382111	12032.6283	1.056897065	0.098308
Csa3G172980	1852.24	1455.093	1615.191	817.5008	748.7838	675.9425	1640.841433	747.4090178	1194.12523	1.133761454	0.103861
Csa7G010800	6884.106	7218.895	7959.49	4360.004	4131.158	4394.727	7354.163672	4295.296279	5824.72998	0.77603609	0.081244
Csa1G391600	153.5645	142.4713	150.7753	38.09143	22.02305	26.42121	148.9370157	28.84523052	88.8911231	2.369526194	0.133808
Csa3G360150	524.8539	420.0808	543.5133	159.2027	214.7248	167.3343	496.1493347	180.4205731	338.284954	1.45780403	0.122493
Csa3G002970	1335.801	1646.801	1529.421	728.6208	478.0838	624.201	1504.007403	610.3018328	1057.15462	1.301951736	0.116648
Csa2G360720	531.1648	553.1239	558.8617	147.4822	74.3278	180.5449	547.7167674	134.1183079	340.917538	2.035457648	0.133094
Csa1G046250	606.8952	480.8406	664.4947	215.8515	154.1614	231.1855	584.0768258	200.3994573	392.238142	1.546858014	0.125669

Csa7G450580	261.901	295.4184	317.8018	105.484	81.66882	110.0884	291.707087	99.0803818	195.393734	1.562679705	0.126498
Csa4G646410	1233.775	1501.187	1379.549	712.9935	757.0424	695.7584	1371.50334	721.9314448	1046.71739	0.92537093	0.096672
Csa2G277620	602.688	711.3089	760.1963	272.5003	293.6407	346.7783	691.3977262	304.306425	497.852076	1.186445968	0.113944
Csa6G301060	327.1134	464.0793	418.9205	162.1328	103.6919	128.8034	403.3710645	131.5426724	267.456868	1.617963668	0.129273
Csa6G511090	213.5177	267.1337	236.5457	79.11298	88.09221	85.86892	239.0656841	84.35803482	161.71186	1.502618374	0.127219
Csa3G610800	787.8068	743.784	768.3219	433.6563	339.5221	337.9713	766.6375603	370.3832106	568.510385	1.049017599	0.108436
Csa3G736630	1429.412	1205.768	1879.725	636.8106	702.9024	626.4027	1504.968404	655.371933	1080.17017	1.199615112	0.117416
Csa3G002330	473.3151	535.3149	648.2434	208.0378	253.2651	243.2953	552.2911717	234.866066	393.578619	1.234783157	0.119264
Csa2G372720	13145.54	13180.69	12885.42	8510.017	7722.75	8935.872	13070.54915	8389.54639	10730.0478	0.639776522	0.078202
Csa7G073710	1564.044	1763.082	1612.483	1033.352	930.4739	934.6501	1646.536281	966.1587765	1306.34753	0.768882484	0.090291
Csa5G633210	375.4967	321.608	471.2856	164.0862	138.5617	144.2157	389.4634309	148.9545422	269.208987	1.388264521	0.125703
Csa5G517880	254.5384	187.5174	148.9696	53.71869	53.22238	49.53976	197.0084416	52.16027504	124.584358	1.913891564	0.133644
Csa2G369080	1180.132	998.3466	1644.083	515.6994	420.2732	566.955	1274.187216	500.9758975	887.581557	1.348425576	0.124907
Csa5G642730	5333.736	6121.028	6240.472	3752.495	3724.649	3620.806	5898.41171	3699.316439	4798.86408	0.673120966	0.083086
Csa5G608500	502.7659	593.9796	538.9991	286.1741	267.9471	225.6811	545.2481734	259.9341235	402.591148	1.066718718	0.113211
Csa6G062290	386.0148	451.5083	564.2787	209.9912	187.1959	179.444	467.2672811	192.2104007	329.738841	1.282380397	0.123226
Csa3G361150	796.2213	673.5959	1065.358	330.1258	387.2387	347.8792	845.0584806	355.0812109	600.069846	1.251458197	0.122226
Csa5G160180	271.3674	283.895	341.2758	121.1112	129.3854	99.07952	298.8460488	116.5253925	207.685721	1.356622919	0.125831
Csa2G000270	1291.624	1322.05	1245.025	751.0849	584.5285	757.4079	1286.232964	697.67378	991.953372	0.883549355	0.103318
Csa3G895630	4175.691	4866.023	5641.885	2563.847	2932.736	2649.827	4894.533114	2715.469896	3805.00151	0.850050953	0.10155
Csa2G036080	801.4803	769.9735	1128.558	384.8212	426.6966	431.5464	900.0037866	414.3547147	657.179251	1.120332212	0.11888
Csa1G045900	405.9992	389.7009	310.579	144.5521	141.3146	181.6458	368.7597116	155.8374935	262.298603	1.244355084	0.124184
Csa6G450430	1295.832	1160.722	1375.937	799.9201	690.0556	718.877	1277.497006	736.2842372	1006.89062	0.795478958	0.0981
Csa6G093640	1314.764	1413.19	1411.148	903.4507	802.0061	732.0876	1379.700711	812.5147978	1096.10776	0.763321578	0.096365
Csa1G050230	116.7511	131.9955	61.39353	12.69714	14.68203	18.71502	103.3800208	15.36473331	59.372377	2.753435599	0.127249
Csa2G006030	497.5068	455.6986	448.7145	265.6633	223.901	201.4617	467.3066334	230.3420186	348.824326	1.018632852	0.115313
Csa7G290540	489.0923	867.3988	528.1649	246.1293	223.901	246.5979	628.2186652	238.8760711	433.547368	1.394917132	0.129826
Csa2G061600	2234.048	2166.402	2741.943	1417.197	1292.019	1446.561	2380.797517	1385.258914	1883.02822	0.782054918	0.099282
Csa1G423280	898.247	823.4003	979.5879	421.9359	328.5105	520.7179	900.4117193	423.7214453	662.066582	1.090120814	0.119916
Csa3G516530	346.046	146.6616	161.6094	49.81188	48.63424	40.73269	218.105675	46.39293567	132.249305	2.229700487	0.131468

Csa3G005590	1181.184	1411.094	1311.835	784.2929	800.1709	662.7319	1301.371285	749.0652184	1025.21825	0.795869828	0.101268
Csa1G002830	1054.967	1155.484	1119.529	675.8788	728.596	645.1178	1109.99334	683.1975059	896.595423	0.699376311	0.092763
Csa2G432230	3151.227	3552.354	3882.238	2073.541	2335.361	2022.323	3528.606333	2143.741882	2836.17411	0.718891643	0.0947
Csa4G022350	2002.649	1978.884	2448.518	1329.293	1095.647	1262.713	2143.350572	1229.217888	1686.28423	0.80289188	0.102374
Csa2G433930	140.9427	156.0899	178.7635	349.6598	301.8993	387.511	158.5987052	346.3567276	252.477716	-1.122975847	0.122653
Csa1G042290	1833.307	1680.323	1423.788	1024.562	938.7326	900.5228	1645.806232	954.6057509	1300.20599	0.784997719	0.101534
Csa1G046040	1467.277	1894.03	1344.338	808.7104	711.1611	910.4307	1568.54827	810.1007358	1189.3245	0.953670084	0.114395
Csa2G349650	874.0553	840.1616	950.6968	563.5579	446.8844	508.6082	888.3045839	506.3501733	697.327379	0.811989504	0.104365
Csa5G635950	315.5434	291.2281	255.5054	140.6453	106.4448	134.3078	287.4256461	127.1326137	207.27913	1.177958494	0.125499
Csa3G895710	149.3572	136.1858	157.9981	271.5236	337.6868	340.173	147.8470237	316.4611248	232.154074	-1.096167707	0.122286
Csa3G841480	415.4655	386.5581	506.4966	214.8748	229.4068	212.4705	436.1734235	218.9173586	327.545391	0.995303047	0.117428
Csa6G510890	1307.402	1046.535	1284.75	676.8555	754.2895	608.7886	1212.895649	679.9778748	946.436762	0.834113847	0.106767
Csa3G149380	21.03623	18.85649	18.95977	81.06639	73.41017	95.77687	19.61749657	83.41780956	51.5176531	-2.087134346	0.131885
Csa6G511000	2653.72	2341.348	2670.618	1642.815	1700.363	1736.093	2555.22895	1693.090567	2124.15976	0.594099203	0.084961
Csa5G598600	242.9684	249.3248	263.631	126.9714	121.1268	105.6848	251.9747469	117.927685	184.951216	1.093711152	0.123059
Csa7G428160	3222.75	3820.535	3617.704	2469.106	2050.897	2111.495	3553.663187	2210.499227	2882.08121	0.684950295	0.095093
Csa1G435760	70.47137	75.42598	55.97645	216.8282	152.3261	182.7467	67.29126605	183.9669814	125.629124	-1.454200654	0.132645
Csa3G865390	4126.256	3246.46	5146.222	1802.018	1342.489	2343.781	4172.979451	1829.429164	3001.20431	1.190311023	0.127334
Csa6G289740	2265.602	2396.87	2907.164	1500.216	1292.019	1606.189	2523.211994	1466.14155	1994.67677	0.784111975	0.104537
Csa6G505270	83.0931	100.568	78.5476	24.41759	14.68203	23.11855	87.40289288	20.73939181	54.0711424	2.081327937	0.131409
Csa3G144190	145.15	212.6594	188.6948	71.29935	69.73966	67.1539	182.1680507	69.39763767	125.782844	1.392103002	0.13208
Csa4G064100	945.5785	802.4486	1200.785	406.3086	559.7526	350.081	982.9374272	438.7140585	710.825743	1.162768949	0.126813
Csa3G878170	1518.816	1663.562	1923.965	1048.003	1092.894	1018.317	1702.114143	1053.071347	1377.59275	0.693007163	0.09763
Csa2G179730	982.3919	891.4932	1148.42	1928.013	1503.073	1892.419	1007.435058	1774.501577	1390.96832	-0.815672908	0.108748
Csa2G007990	308.1808	318.4652	358.4299	168.9697	179.8549	135.4087	328.3586205	161.4110993	244.88486	1.022135018	0.121915
Csa5G152910	575.3409	516.4584	643.7292	296.9178	332.181	348.9801	578.5094999	326.0263231	452.267912	0.829044174	0.111023
Csa3G914040	927.6977	800.3535	931.7371	459.0506	566.176	546.0382	886.596071	523.7549418	705.175506	0.759738114	0.105549
Csa1G025040	305.0253	404.3671	322.316	183.6202	167.0081	166.2334	343.9027991	172.2872696	258.095034	0.996290075	0.121879
Csa6G519630	355.5123	393.8912	397.2522	119.1578	221.1481	144.2157	382.218578	161.5072379	271.862908	1.240086878	0.130735

Csa5G576720	4289.287	3397.312	5315.957	2223.954	2608.814	2333.873	4334.185378	2388.880306	3361.53284	0.859549054	0.114279
Csa1G043040	1661.862	1672.99	1812.915	1180.834	1210.35	1164.735	1715.922325	1185.3065	1450.61441	0.533888114	0.083458
Csa2G000800	96.76665	147.7092	62.29637	12.69714	25.69356	18.71502	102.2574122	19.03524199	60.6463271	2.418610835	0.125006
Csa2G372820	57.84963	49.2364	56.8793	157.2493	165.1729	116.6937	54.6551102	146.3719338	100.513522	-1.422913814	0.133375
Csa3G203270	440.709	360.3686	313.2876	203.1543	167.0081	178.3431	371.4550417	182.8351989	277.14512	1.021495759	0.12389
Csa6G401410	87.30035	169.7085	83.96468	24.41759	22.02305	31.92562	113.6578273	26.12208709	69.8899572	2.125493798	0.128889
Csa7G440550	245.0721	208.469	126.3984	76.18287	58.72814	55.04418	193.3131776	63.31839499	128.315786	1.606391566	0.133716
Csa6G487780	2623.218	2934.28	2829.519	1962.197	2005.015	1971.682	2795.672407	1979.631676	2387.65204	0.497971944	0.079974
Csa6G290280	1043.397	958.5385	961.531	610.4396	688.2204	639.6134	987.8221434	646.0911248	816.956634	0.611852157	0.094146
Csa1G042560	470.1597	560.4569	559.7645	816.5241	865.3224	844.3777	530.1270553	842.0747285	686.100892	-0.66695714	0.099952
Csa3G607630	3072.341	3186.748	3772.091	1933.873	2373.901	1960.674	3343.726482	2089.482638	2716.60456	0.678232687	0.101084
Csa2G271390	820.4129	878.9222	956.1139	1293.155	1327.807	1344.179	885.1496723	1321.713564	1103.43162	-0.577516013	0.090419
Csa3G116740	6120.491	6750.625	8449.736	4260.38	4558.772	4465.184	7106.950554	4428.111965	5767.53126	0.68270533	0.101663
Csa5G262260	5162.291	5405.529	6147.478	7823.395	7881.5	8265.434	5571.765835	7990.109356	6780.9376	-0.519830335	0.084209
Csa1G207820	6686.365	7057.567	6767.734	5092.532	3996.266	4638.022	6837.221985	4575.606829	5706.41441	0.579554154	0.091525
Csa5G182750	543.7865	596.0748	594.0727	356.4968	375.3095	304.9447	577.9779795	345.5836711	461.780825	0.740291748	0.107672
Csa5G589890	1784.924	1778.796	1888.754	1192.555	1187.41	1354.087	1817.491289	1244.683745	1531.08752	0.547099062	0.087776
Csa2G370560	709.9727	1312.622	834.2297	317.4286	518.4594	282.9271	952.2746629	372.9383476	662.606505	1.350703806	0.13332
Csa4G308640	695.2474	543.6956	552.5418	316.4519	267.0295	373.1995	597.1615725	318.8936491	458.027611	0.906800519	0.119927
Csa4G628870	134.6319	133.043	193.209	55.6721	62.39865	60.5486	153.6279853	59.53977977	106.583883	1.370304784	0.133446
Csa1G269880	4720.53	3860.344	4337.272	5903.196	6671.15	6528.24	4306.04849	6367.528205	5336.78835	-0.564356791	0.090448
Csa1G574980	262.9529	304.8467	395.4465	84.9732	174.3492	103.4831	321.0820253	120.9351387	221.008582	1.406444755	0.133774
Csa6G430140	4778.379	4286.71	5748.42	2599.008	3475.054	2551.848	4937.836566	2875.303344	3906.56996	0.78003346	0.112801
Csa4G642290	237.7094	184.3746	236.5457	75.20616	71.57492	122.1981	219.5432181	89.65971974	154.601469	1.299125833	0.133179
Csa3G038090	59.95325	107.9011	120.0785	31.25451	16.51729	11.00884	95.9776081	19.59354487	57.7855765	2.286914533	0.123627
Csa3G112250	2067.861	1094.724	2484.632	293.011	203.7132	420.5375	1882.405924	305.7539278	1094.07993	2.622925593	0.117848
Csa6G494990	2133.074	2042.787	1691.031	1344.921	1040.589	1235.191	1955.630369	1206.900399	1581.26538	0.69627835	0.106495

Csa1G046260	1154.889	1047.583	983.1993	677.8322	464.3193	670.4381	1061.890435	604.1965406	833.043488	0.814465336	0.116494
Csa5G190560	254.5384	313.2273	301.5506	143.5754	137.6441	175.0405	289.7720882	152.0866556	220.929372	0.933577103	0.123806
Csa5G160200	1137.008	1174.341	1138.489	750.1082	810.2648	835.5706	1149.945858	798.6478844	974.296871	0.526231322	0.088825
Csa1G050570	607.947	568.8376	626.5751	299.848	278.9587	418.3358	601.119911	332.3807894	466.75035	0.85797231	0.119993
Csa2G360620	517.4912	510.1729	696.9971	203.1543	358.7922	192.6546	574.8870954	251.5337204	413.210408	1.190607675	0.132319
Csa3G184590	325.0097	345.7024	323.2189	546.9539	492.7658	527.3232	331.3103366	522.3476468	426.828992	-0.656864271	0.104033
Csa3G184580	570.0818	481.8882	492.9539	796.99	755.2072	810.2503	514.97464	787.4824887	651.228564	-0.612963399	0.100057
Csa4G647490	265.0565	201.1359	253.6997	475.6546	414.7675	389.7128	239.9640516	426.7116116	333.337832	-0.830206093	0.118875
Csa6G085120	158.8235	192.7553	136.3297	326.2189	282.6292	311.55	162.6361849	306.799388	234.717787	-0.917974991	0.123999
Csa6G449290	849.8636	992.0611	695.1914	513.746	496.4363	506.4064	845.7054043	505.5295816	675.617493	0.741454715	0.112521
Csa5G608450	1042.345	1230.91	1157.449	730.5742	703.82	827.8644	1143.567931	754.086216	948.827074	0.601815148	0.099188
Csa3G778430	100.9739	75.42598	110.1472	31.25451	36.70509	30.82474	95.51569664	32.92811216	64.2219044	1.535574098	0.133161
Csa2G326480	402.8438	413.7953	542.6104	242.2225	247.7593	278.5235	453.0831775	256.1684431	354.62581	0.825474507	0.119645
Csa6G423990	1884.846	1810.224	2242.669	1399.616	1130.517	1322.161	1979.246359	1284.097952	1631.67216	0.624969703	0.102864
Csa6G046400	1147.526	1489.663	1204.397	1851.83	1960.969	2001.406	1280.528648	1938.068431	1609.29854	-0.598091535	0.100015
Csa6G037500	875.1071	671.5007	838.7439	1383.012	1446.18	1101.984	795.1172626	1310.39231	1052.75479	-0.721098256	0.112064
Csa3G857030	714.18	627.5022	744.8479	404.3552	465.237	299.4403	695.5100551	389.6775088	542.593782	0.83378178	0.120388
Csa3G016990	713.1282	733.3081	660.8833	968.8898	1027.742	1037.032	702.4398547	1011.221518	856.830686	-0.526237826	0.091663
Csa6G501990	1465.173	1461.378	2275.172	955.216	1009.39	977.5846	1733.907871	980.73015	1357.31901	0.822695782	0.119811
Csa3G852470	1106.506	694.5476	1155.643	571.3715	530.3885	407.3269	985.5653593	503.0289773	744.297168	0.969840656	0.127433
Csa2G007390	1759.681	2057.453	1900.491	1381.059	1102.988	1316.657	1905.874827	1266.901086	1586.38796	0.589608249	0.099344
Csa6G085110	755.2006	488.1737	838.7439	1280.458	1313.124	1190.055	694.0394166	1261.212605	977.626011	-0.860957926	0.122388
Csa3G903530	144.0982	120.4721	117.37	250.0361	224.8187	233.3873	127.3133994	236.0806838	181.697042	-0.892535191	0.124168
Csa2G286540	260.8492	292.2757	217.5859	88.88001	142.2322	132.106	256.9035981	121.0727505	188.988174	1.08413762	0.131218
Csa1G575060	135.6837	142.4713	112.8558	63.48572	54.14	51.74153	130.3369069	56.45575125	93.3963291	1.203831449	0.133181
Csa4G015820	1356.837	1626.896	1578.175	1075.35	1071.789	968.7775	1520.636015	1038.638851	1279.63743	0.549680822	0.095457

Csa4G378260	579.5481	467.222	465.8685	301.8014	322.0871	232.2864	504.2128934	285.3916433	394.802268	0.818089887	0.120445
Csa7G432080	49.43514	59.71223	76.74191	9.767034	6.42339	20.91679	61.96309386	12.36907075	37.1660823	2.350764411	0.119463
Csa3G853150	27.3471	35.61782	35.21099	95.71694	99.10373	75.96097	32.72530506	90.26054561	61.4929253	-1.463780851	0.133219
Csa6G383000	761.5115	640.0732	720.4711	464.9108	472.578	472.279	707.3519449	469.9226253	588.637285	0.590187104	0.100822
Csa6G511670	2400.234	2479.629	2443.101	3332.512	3086.898	3278.431	2440.988021	3232.613723	2836.80087	-0.405095825	0.076094
Csa5G583370	993.9618	791.9728	810.7557	1380.082	1329.642	1215.375	865.5634352	1308.366392	1086.96491	-0.596875138	0.101806
Csa2G379980	136.7355	103.7107	272.6595	56.6488	55.05763	50.64064	171.0352337	54.11569111	112.575462	1.662454838	0.130428
Csa1G575000	273.471	380.2726	371.0697	574.3016	536.8119	608.7886	341.6044426	573.3007082	457.452575	-0.745352622	0.116254
Csa3G710780	2134.125	1920.22	3065.162	1501.193	1174.563	1395.92	2373.169105	1357.225438	1865.19727	0.806775271	0.120763
Csa6G087740	1385.236	1440.427	1206.202	976.7034	823.1116	922.5404	1343.954873	907.4518107	1125.70334	0.566442402	0.09915
Csa3G116630	226.1395	218.9449	299.7449	143.5754	121.1268	121.0972	248.2763964	128.5997944	188.438095	0.950812957	0.128213
Csa5G609720	1943.748	1454.045	1839.097	1220.879	1127.764	1084.37	1745.630039	1144.337798	1444.98392	0.609210156	0.104448
Csa2G264590	236.6576	437.8897	244.6713	116.2277	135.8088	151.9219	306.4061851	134.6528205	220.529503	1.186319215	0.133526
Csa3G149300	36.8134	27.23716	18.95977	1.953407	0.917627	1.100884	27.67010858	1.323972534	14.4970406	4.381838941	0.097779
Csa1G257350	698.4028	681.9766	784.5732	491.2818	490.9305	495.3976	721.6508455	492.5366557	607.093751	0.552035339	0.098767
Csa3G733180	574.289	565.6948	558.8617	366.2638	290.8878	399.6207	566.2818559	352.2574448	459.26965	0.687062321	0.112937
Csa6G507480	135.6837	169.7085	198.6261	59.57891	91.76272	73.7592	168.0060833	75.03360841	121.519846	1.16274534	0.133439
Csa3G132580	170.3935	122.5672	135.4269	59.57891	75.24543	57.24595	142.7958568	64.02342758	103.409642	1.15298073	0.133351
Csa2G238790	427.0354	523.7915	462.2572	660.2515	735.0194	745.2982	471.0280407	713.5230191	592.27553	-0.59912681	0.104749
Csa5G158580	2514.881	2337.158	3030.854	3933.185	3609.028	3850.891	2627.630987	3797.701032	3212.66601	-0.530868563	0.096756
Csa5G623420	1526.178	1733.75	1530.324	1185.718	1057.106	1165.836	1596.750773	1136.220054	1366.48541	0.491054798	0.09159
Csa2G405050	1195.91	1143.961	1145.712	776.4792	891.9336	822.36	1161.860624	830.2576205	996.059122	0.484289287	0.090886
Csa4G652640	69.41956	71.23565	57.78214	17.58066	15.59966	27.52209	66.14578217	20.2341376	43.1899599	1.719130984	0.12861
Csa2G425760	1567.199	1455.093	2079.254	1029.445	1161.716	1030.427	1700.515414	1073.862811	1387.18911	0.663495148	0.111587
Csa1G169940	185.1188	131.9955	180.5692	83.01979	83.50407	51.74153	165.8944926	72.75513063	119.324812	1.184361424	0.133678
Csa3G840430	136.7355	106.8535	132.7184	66.41583	34.86983	49.53976	125.4357735	50.27514213	87.8554578	1.32208159	0.133592
Csa2G000030	2795.715	2483.819	3142.807	2060.844	1953.628	1935.353	2807.447049	1983.275265	2395.36116	0.501662031	0.093794
Csa1G231030	76.78224	21.99924	36.11384	4.883517	4.588136	5.504418	44.96510641	4.992023613	24.978565	3.173827223	0.104609
Csa3G374190	1059.174	1477.092	812.5614	636.8106	611.1397	610.9904	1116.275874	619.6469041	867.961389	0.84845661	0.125322
Csa4G378780	338.6833	410.6526	273.5623	189.4805	132.1383	200.3608	340.9660592	173.9931958	257.479628	0.971984344	0.130316
Csa3G176320	4833.074	5696.757	5432.424	3214.331	3921.021	3990.703	5320.751527	3708.684939	4514.71823	0.520837994	0.096655

Csa4G642510	1475.691	1468.711	1654.917	1144.696	816.6882	1032.629	1533.106535	998.0044631	1265.5555	0.62015133	0.108301
Csa1G324370	198.7924	169.7085	215.7802	346.7297	288.1349	416.134	194.7603368	350.3328792	272.546608	-0.843594286	0.12532
Csa7G387690	1060.226	1227.767	1041.884	1711.184	1422.322	1793.339	1109.959184	1642.281952	1376.12057	-0.565016125	0.102405
Csa1G014530	78.88586	72.28323	70.42199	29.3011	19.27017	29.72386	73.86369217	26.09837661	49.9810344	1.506856904	0.131532
Csa5G599810	909.8169	925.0158	890.2062	653.4146	673.5383	646.2187	908.3462952	657.723864	783.03508	0.465215428	0.089488
Csa4G630010	529.0612	493.4116	506.4966	307.6616	368.8861	329.1642	509.6564591	335.2372965	422.446878	0.60337837	0.107029
Csa2G406800	113.5956	120.4721	79.45045	40.04484	31.19932	49.53976	104.5060448	40.26130832	72.3836766	1.380070504	0.132952
Csa6G151130	993.9618	1118.819	1203.494	406.3086	414.7675	816.8556	1105.424742	545.9772378	825.70099	1.019636215	0.131915
Csa1G526840	2134.125	2030.216	2030.501	3332.512	3182.331	2500.107	2064.94734	3004.983239	2534.96529	-0.541680978	0.100333
Csa1G014370	956.0966	1087.391	1003.965	761.8287	640.5038	715.5743	1015.817519	705.9689199	860.89322	0.525380169	0.098359
Csa4G639800	100.9739	103.7107	81.25614	234.4088	145.9027	221.2776	95.31358676	200.5297135	147.92165	-1.073761487	0.1329
Csa3G147770	2482.275	2671.337	2993.837	2079.402	1943.534	1859.392	2715.816371	1960.776099	2338.29624	0.470122792	0.091151
Csa3G016400	2486.482	2903.9	2373.582	3744.681	3319.975	4099.69	2587.988198	3721.44882	3154.71851	-0.524005356	0.098501
Csa2G359890	28.39891	20.95166	44.23945	129.9016	68.82204	96.87775	31.19667463	98.53378272	64.8652287	-1.649496402	0.1284
Csa3G710790	1862.758	1543.09	2385.319	1281.435	947.9089	1228.586	1930.389009	1152.643277	1541.51614	0.744754353	0.12033
Csa2G381760	5066.576	4012.243	4719.176	7294.998	7207.961	5768.63	4599.331611	6757.196415	5678.26401	-0.555109382	0.102718
Csa1G062380	199.8442	169.7085	201.3347	99.62375	95.43323	115.5928	190.295762	103.5499167	146.922839	0.881544798	0.128087
Csa6G501230	3912.739	2830.569	2912.581	2335.298	1726.057	2024.525	3218.629719	2028.626497	2623.62811	0.665932094	0.11442
Csa2G007960	222.984	305.8943	204.946	148.4589	108.28	115.5928	244.6081061	124.1105674	184.359337	0.977402872	0.131332
Csa3G143540	980.2883	776.259	1244.122	616.2999	610.2221	603.2842	1000.22303	609.935376	805.079203	0.714442824	0.118859
Csa4G432970	322.9061	108.9486	269.951	52.74199	110.1153	46.23711	233.9352345	69.6981185	151.816677	1.742692978	0.125682
Csa2G361810	1139.112	1132.437	1320.864	1574.446	1643.47	1781.23	1197.470922	1666.381934	1431.92643	-0.475708309	0.093513
Csa1G042780	311.3362	567.79	395.4465	240.269	230.3244	183.8476	424.8575826	218.1470066	321.502295	0.959991248	0.131221
Csa2G382550	464.9007	380.2726	392.738	272.5003	212.8895	271.9182	412.6371047	252.4360006	332.536553	0.710277013	0.118999
Csa7G388340	116.7511	114.1866	109.2444	193.3873	182.6078	236.69	113.3939961	204.228351	158.811174	-0.847261507	0.127458
Csa6G404200	23.13985	15.71375	24.37684	0	0	1.100884	21.07681315	0.366961188	10.7218872	5.886605681	0.088656
Csa3G560210	1363.148	1073.773	1492.404	886.8467	912.1214	804.7459	1309.774897	867.9046701	1088.83978	0.593776635	0.108948
Csa1G537570	133.5801	91.13973	92.09029	43.95165	50.46949	38.53092	105.6033568	44.31735787	74.9603573	1.247113238	0.133543

Csa4G639080	589.0144	634.8353	604.004	914.1944	829.535	809.1494	609.2845698	850.9595985	730.122084	-0.482358355	0.095508
Csa2G264570	1821.737	1622.706	2849.382	1281.435	1147.034	1253.906	2097.941845	1227.458416	1662.70013	0.773921511	0.124161
Csa5G099490	186.1706	173.8988	149.8724	97.67034	66.06916	94.67599	169.9806158	86.13849539	128.059556	0.982797133	0.132198
Csa1G713660	648.9677	645.3112	772.8362	1013.818	947.9089	968.7775	689.0383313	976.8348558	832.936594	-0.502265111	0.098627
Csa3G016920	4266.147	3804.822	4466.379	5861.197	8718.376	5505.519	4179.116001	6695.030584	5437.07329	-0.679988042	0.117667
Csa5G190520	206.155	162.3754	200.4318	104.5073	73.41017	113.391	189.6540757	97.10281604	143.378446	0.970743811	0.131993
Csa5G611040	375.4967	427.4139	352.1099	239.2923	235.8302	266.4138	385.0068363	247.1787822	316.092809	0.639736302	0.114538
Csa1G002810	611.1024	614.9313	844.161	335.0093	404.6736	487.6914	690.0649025	409.1247595	549.594831	0.756413112	0.123678
Csa5G638460	353.4086	299.6088	318.7046	205.1077	214.7248	199.2599	323.9073448	206.3641347	265.13574	0.649280223	0.115641
Csa5G375760	944.5267	766.8308	1092.444	586.9988	654.2682	592.2754	934.6003755	611.1807645	772.89057	0.613183034	0.112421
Csa4G006040	32.60615	68.0929	54.17076	16.60396	8.258645	13.2106	51.62327098	12.69106856	32.1571698	2.030165194	0.117958
Csa6G507230	424.9318	415.8905	535.3877	300.8247	296.3936	291.7341	458.736657	296.3174593	377.527058	0.631968921	0.114503
Csa5G601520	323.9579	300.6563	302.4534	153.3424	160.5848	226.782	309.0225564	180.2364027	244.62948	0.781485038	0.125527
Csa6G190340	2191.975	2358.109	2415.113	1596.91	1856.36	1761.414	2321.732513	1738.22786	2029.98019	0.417669782	0.087642
Csa4G652740	411.2583	314.2749	421.6291	140.6453	261.5237	199.2599	382.3874235	200.476321	291.431872	0.930903032	0.131555
Csa2G035330	3019.751	2606.387	3598.744	2042.287	2212.399	2169.842	3074.960477	2141.509164	2608.23482	0.522278192	0.10273
Csa3G732560	1693.416	1726.417	1673.876	1325.387	1338.818	1275.924	1697.903257	1313.376217	1505.63974	0.370074059	0.079957
Csa1G073810	407.051	255.6103	265.4367	573.3249	558.8349	471.1782	309.3660053	534.4460092	421.906007	-0.790624648	0.126306
Csa5G157930	2240.358	2004.026	2092.797	1604.724	1682.928	1467.478	2112.393924	1585.043254	1848.71859	0.413772495	0.087797
Csa1G499320	418.621	534.2674	529.0678	309.615	352.3688	256.5059	493.985355	306.1632308	400.074293	0.688525309	0.120141
Csa5G290670	155.6681	206.3739	279.8823	99.62375	93.59797	123.299	213.974738	105.5068936	159.740816	1.025236915	0.133386
Csa6G511080	151.4608	231.5159	125.4956	88.88001	72.49255	72.65832	169.4907652	78.01029139	123.750528	1.116854522	0.133819
Csa6G517220	9157.07	7728.02	14398.59	6061.422	5957.236	6579.981	10427.89286	6199.546053	8313.71946	0.750352831	0.124437
Csa6G011040	270.3155	336.2742	291.6193	177.76	199.1251	182.7467	299.4029858	186.5439308	242.973458	0.681468749	0.119662
Csa6G141420	903.506	584.5513	847.7724	566.488	370.7214	357.7872	778.6099212	431.665509	605.137715	0.850782716	0.129407
Csa5G139070	479.626	411.7001	655.4662	229.5253	340.4397	296.1377	515.5974506	288.7008885	402.14917	0.837530811	0.128888
Csa1G617380	376.5485	320.5604	387.3209	229.5253	253.2651	199.2599	361.4766144	227.3501103	294.413362	0.667400526	0.118669
Csa1G038950	114.6474	160.2802	140.844	74.22946	74.3278	47.33799	138.5905432	65.29841834	101.944481	1.080272277	0.133779
Csa4G043890	3171.211	2722.668	3349.559	2272.789	2297.738	2336.075	3081.146167	2302.200749	2691.67346	0.420761282	0.089902

Csa2G369020	3776.003	3259.031	4733.622	2294.276	3002.476	2394.422	3922.885173	2563.724739	3243.30496	0.613690816	0.114414
Csa3G176310	257.6938	313.2273	280.7851	169.9464	176.1844	189.352	283.9020803	178.4942624	231.198171	0.670469371	0.119519
Csa3G146310	689.9883	450.4607	863.1208	285.1974	291.8054	453.564	667.8565975	343.5222903	505.689444	0.961563205	0.132772
Csa5G548130	1846.981	1875.174	1645.888	1299.016	1337.9	1415.736	1789.347603	1350.884081	1570.11584	0.405402191	0.088015
Csa7G398090	5445.228	4749.742	4589.166	3343.256	3999.019	3599.889	4928.045199	3647.388105	4287.71665	0.433916792	0.092998
Csa3G009510	3003.973	2052.215	2842.159	1857.69	1812.314	1644.72	2632.782626	1771.574546	2202.17859	0.571504984	0.111082
Csa6G504590	195.6369	190.6601	182.3749	113.2976	78.91594	121.0972	189.5573117	104.436909	146.99711	0.863970098	0.130679
Csa1G039920	278.73	221.04	231.1286	134.7851	163.3376	137.6104	243.6328771	145.2443852	194.438631	0.743804389	0.125384
Csa7G257840	409.1547	422.176	463.16	653.4146	552.4116	668.2363	431.4968726	624.6874923	528.092183	-0.531897982	0.106716
Csa2G296130	1995.286	1686.609	1994.387	1469.939	1276.419	1420.14	1892.093943	1388.83262	1640.46328	0.446616775	0.095221
Csa6G190370	1836.463	1502.234	1700.059	2291.346	2645.519	2109.293	1679.585296	2348.719432	2014.15236	-0.484122945	0.100709
Csa4G641760	327.1134	203.2311	256.4083	173.8532	116.5387	123.299	262.2509113	137.8969404	200.073926	0.926834549	0.132444
Csa5G623890	253.4866	184.3746	343.9843	110.3675	160.5848	69.35566	260.6151667	113.4359691	187.025568	1.196848654	0.133062
Csa3G732510	1919.556	2187.353	2357.331	1620.351	1573.731	1640.317	2154.746731	1611.466035	1883.10638	0.419672185	0.091384
Csa7G009780	892.9879	751.117	1116.821	632.9038	609.3044	571.3586	920.3084874	604.5222786	762.415383	0.606966753	0.11527
Csa4G644730	400.7402	435.7945	368.3612	267.6167	289.9702	230.0847	401.6319586	262.5571972	332.094578	0.610155563	0.115598
Csa5G160190	45.22789	58.66465	49.65653	22.46418	11.01153	14.31149	51.18302426	15.92906379	33.556044	1.685055445	0.12326
Csa4G645820	313.4398	205.3263	295.2306	147.4822	161.5024	160.729	271.3322431	156.5712003	213.951722	0.794157949	0.128343
Csa2G258750	74.67861	73.33081	28.89107	13.67385	17.43492	16.51325	58.96683261	15.87400592	37.4204193	1.888316969	0.118153
Csa3G026150	5607.207	6793.576	6563.69	8234.587	9508.453	7948.379	6321.491097	8563.806239	7442.64867	-0.438033946	0.094515
Csa6G504600	388.1184	483.9834	491.1482	310.5917	312.9109	280.7253	454.4166716	301.4092886	377.91298	0.592112824	0.114101
Csa1G045730	1151.734	1102.057	1273.013	1635.978	1441.592	1744.9	1175.601252	1607.490328	1391.54579	-0.450351271	0.096589
Csa2G379090	49.43514	90.09214	60.49068	12.69714	29.36407	20.91679	66.67265402	20.99266726	43.8326606	1.662309109	0.123426
Csa2G382570	335.5278	290.1805	295.2306	198.2708	194.537	209.1679	306.9796655	200.6585448	253.819105	0.613752624	0.116242
Csa1G561930	804.6358	1023.489	979.5879	1282.412	1240.632	1416.837	935.9041008	1313.293563	1124.59883	-0.487980133	0.101953
Csa6G495620	693.1437	700.8331	781.8646	502.0256	540.4824	538.3321	725.2804787	526.946677	626.113578	0.461772357	0.098401
Csa5G153070	1188.547	992.0611	988.6164	1589.096	1302.113	1583.071	1056.408149	1491.426669	1273.91741	-0.497481571	0.103397
Csa2G356090	2545.384	2650.385	2572.208	3663.615	3119.015	5062.964	2589.325686	3948.530947	3268.92832	-0.608413913	0.11605

Csa7G433230	64.1605	39.80816	56.8793	10.74374	23.85831	12.10972	53.61598393	15.5705878	34.5932859	1.773365418	0.120457
Csa1G056950	465.9525	460.9365	531.7763	769.6423	681.797	633.008	486.2217681	694.8157809	590.518775	-0.514504055	0.10589
Csa3G081900	189.3261	119.4245	258.214	71.29935	110.1153	53.94329	188.988161	78.45263523	133.720398	1.26543751	0.131737
Csa3G842010	734.1644	925.0158	602.1983	509.8392	463.4017	485.4897	753.7928337	486.2435217	620.018178	0.631622705	0.118577
Csa3G153170	642.6568	617.0264	737.6252	523.513	403.756	422.7393	665.769463	450.0027614	557.886112	0.565770538	0.112205
Csa3G904070	240.8648	218.9449	314.1904	416.0757	367.9685	468.9764	258.0000285	417.6735188	337.836774	-0.691302399	0.123258
Csa6G423410	390.222	384.463	373.7782	226.5952	295.4759	213.5714	382.8210893	245.2141856	314.017638	0.639670009	0.119299
Csa4G436980	397.5847	532.1722	536.2905	288.1275	334.9339	329.1642	488.6824794	317.4085385	403.045509	0.623465879	0.118106
Csa5G175170	653.1749	679.8814	506.4966	896.6138	814.8529	999.6023	613.1843035	903.6896518	758.436978	-0.560131953	0.112099
Csa2G155100	264.0047	278.6571	309.6762	547.9306	371.639	435.9499	284.112647	451.8398406	367.976244	-0.668010907	0.122013
Csa6G518360	1178.029	1171.198	1082.512	853.6388	890.0984	882.9086	1143.913006	875.5485915	1009.7308	0.385248302	0.087729
Csa3G300600	840.3973	705.0234	790.8931	1197.438	1158.045	947.8607	778.7712767	1101.114882	939.94308	-0.500334569	0.105147
Csa5G188900	118.8547	101.6156	115.5643	70.32265	44.0461	48.43888	112.0115122	54.26920942	83.1403608	1.045597752	0.133817
Csa5G217170	17.88079	8.380664	17.15407	46.88176	43.12848	49.53976	14.4718443	46.5166674	30.4942559	-1.677818397	0.121833
Csa2G297220	34.70978	28.28474	26.18253	101.5772	44.96373	104.5839	29.72568463	83.70827569	56.7169802	-1.49265649	0.126286
Csa3G116640	1850.136	1359.763	2154.191	1160.324	1301.195	975.3828	1788.029893	1145.633948	1466.83192	0.642076617	0.120508
Csa3G036410	886.677	933.3965	851.3838	1150.557	1128.681	1206.568	890.4857735	1161.935483	1026.21063	-0.383958278	0.088094
Csa3G398920	44.17608	43.99849	45.1423	9.767034	17.43492	15.41237	44.43895621	14.20477348	29.3218649	1.644834757	0.122523
Csa4G378770	780.4441	931.3013	777.3504	654.3913	543.2353	444.757	829.6986089	547.4611818	688.579895	0.598436774	0.116814
Csa3G002940	529.0612	601.3127	581.4328	427.7961	391.8268	412.8313	570.6022165	410.8180807	490.710149	0.474565552	0.10253
Csa6G110860	3331.087	2961.517	3577.076	4117.782	4432.139	4254.915	3289.893323	4268.278628	3779.08598	-0.375397003	0.087126
Csa1G038960	89.40397	83.80664	106.5358	39.06814	53.22238	22.01767	93.24881483	38.10272817	65.6757715	1.282122856	0.130752
Csa3G842100	3846.474	3828.916	3181.629	2605.845	2811.61	2817.161	3619.006597	2744.871817	3181.93921	0.398596662	0.091142
Csa3G734210	1251.656	1359.763	1327.184	1696.534	1628.788	1686.554	1312.867345	1670.625237	1491.74629	-0.347433039	0.082178
Csa4G056500	3249.046	2064.786	4173.857	1519.751	2094.943	1921.042	3162.56293	1845.245064	2503.904	0.777488393	0.129259
Csa3G144200	23.13985	25.14199	46.04515	7.813627	3.670509	6.605301	31.4423303	6.029812513	18.7360714	2.39657625	0.104842
Csa6G502710	2126.763	2331.92	2330.246	1735.602	1804.055	1823.063	2262.976043	1787.5734	2025.27472	0.340470648	0.081203

Csa4G167980	2956.642	3009.706	3419.078	4165.64	4079.77	3809.057	3128.475288	4018.155892	3573.31559	-0.360892194	0.085165
Csa1G015080	1850.136	1800.795	2460.255	1318.55	1527.849	1438.855	2037.062313	1428.417897	1732.74011	0.512529733	0.108457
Csa7G046690	2957.694	2962.565	2620.962	2069.635	2037.132	2394.422	2847.073525	2167.062882	2507.0682	0.393944728	0.091407
Csa1G071850	96.76665	77.52115	106.5358	52.74199	37.62271	44.03534	93.60787542	44.80001399	69.2039447	1.066305457	0.133562
Csa1G570740	391.2739	404.3671	452.3258	758.8986	474.4132	770.6185	415.9889198	667.97677	541.982845	-0.681587617	0.125265
Csa4G642520	5881.73	5665.329	6818.293	4772.173	4817.543	4786.642	6121.783901	4792.119119	5456.95151	0.35347611	0.085557
Csa1G103250	194.5851	185.4222	146.2611	119.1578	73.41017	94.67599	175.4227895	95.74799303	135.585391	0.873249966	0.13293
Csa1G154090	437.5536	404.3671	607.6154	204.131	371.639	164.0317	483.1786582	246.6005575	364.889608	0.968445119	0.133812
Csa3G751450	723.6463	613.8837	678.0373	859.499	958.9204	881.8077	671.8557616	900.0757164	785.965739	-0.422178239	0.09793
Csa6G190490	473.3151	463.0317	492.0511	339.8928	299.1465	372.0986	476.1326422	337.0459657	406.589304	0.500749814	0.109085
Csa6G526420	227.1913	144.5665	240.157	113.2976	100.939	125.5007	203.9715894	113.2457711	158.60868	0.852488428	0.132671
Csa3G710870	3881.184	2352.872	3434.426	2222.977	2228.916	1290.236	3222.82731	1914.042983	2568.43515	0.75134457	0.129773
Csa6G497220	6302.454	7864.206	6512.228	5617.998	5016.668	5101.494	6892.96277	5245.386776	6069.17477	0.39400483	0.093966
Csa1G231010	534.3202	661.0249	580.53	442.4467	392.7444	434.849	591.9583649	423.3466974	507.652531	0.484150463	0.10761
Csa1G062300	204.0514	186.4698	178.7635	93.76353	122.962	127.7025	189.7615702	114.8093546	152.285462	0.725396463	0.12872
Csa3G280950	1464.122	1369.191	1653.111	1118.325	1113.999	1193.358	1495.474533	1141.8942	1318.68437	0.390100142	0.09349
Csa3G149910	645.8122	503.8874	512.8165	384.8212	389.9915	393.0154	554.1720666	389.2760442	471.724055	0.508921688	0.11089
Csa4G214830	1965.836	1763.082	1715.407	1517.797	1226.868	1350.784	1814.775085	1365.149599	1589.96234	0.41067185	0.097021
Csa4G075200	1695.52	1218.339	2132.522	1017.725	1216.774	1012.813	1682.127127	1082.437161	1382.28214	0.63619587	0.12342
Csa4G152270	589.0144	395.9864	491.1482	295.9411	274.3705	370.9978	492.0496746	313.7698084	402.909742	0.650920121	0.124551
Csa3G359120	2630.58	2016.597	2834.034	3389.161	4544.09	3162.838	2493.737126	3698.69638	3096.21675	-0.568726909	0.117728
Csa3G748260	3975.847	3695.873	4790.501	3149.869	2850.15	3305.953	4154.073708	3101.990636	3628.03217	0.421785357	0.099203
Csa7G061200	1883.794	2426.202	2118.077	1351.758	1674.67	1650.224	2142.691117	1558.883867	1850.78749	0.458960554	0.104897
Csa1G051590	2210.908	2426.202	2451.227	1996.382	1804.973	1776.826	2362.778961	1859.393512	2111.08624	0.345683453	0.086098
Csa1G024910	154.6163	180.1843	197.7233	122.0879	102.7742	92.47422	177.5079468	105.7787972	141.643372	0.746563277	0.13006

Csa6G390120	293.4554	302.7515	233.8371	170.9231	90.84509	187.1502	276.6813358	149.6394656	213.160401	0.889556562	0.133536
Csa5G496510	282.9373	218.9449	283.4936	384.8212	374.3919	388.6119	261.7919263	382.6083121	322.200119	-0.545995804	0.11596
Csa3G640640	820.4129	668.358	1151.129	430.7262	659.7739	504.2047	879.9665199	531.5682739	705.767397	0.72740131	0.129364
Csa6G524030	52.59057	37.71299	45.1423	13.67385	15.59966	19.8159	45.14862061	16.36313804	30.7558793	1.469997423	0.123577
Csa1G506960	150.409	113.139	108.3415	49.81188	69.73966	74.86008	123.9631752	64.80387412	94.3835246	0.936107573	0.133807
Csa1G533380	28.39891	20.95166	23.474	63.48572	63.31627	47.33799	24.2748553	58.04666373	41.1607595	-1.261701918	0.129125
Csa2G377910	161.979	148.7568	157.0952	105.484	78.91594	94.67599	155.943653	93.02529801	124.484476	0.747116056	0.13028
Csa7G044910	319.7507	298.5612	353.9156	243.1992	191.7841	219.0758	324.0758264	218.0196876	271.047757	0.573814025	0.119092
Csa2G037230	5292.715	4962.401	5324.083	3807.19	4554.184	3821.167	5193.066314	4060.846827	4626.95657	0.354619447	0.088609
Csa2G361700	2859.875	3631.97	3515.682	2526.732	2528.063	2602.489	3335.842689	2552.427794	2944.13524	0.386383855	0.094328
Csa6G188110	820.4129	867.3988	835.1325	1168.137	973.6024	1313.354	840.9814131	1151.697942	996.339678	-0.452729054	0.104914
Csa7G447020	3109.155	2709.05	4049.264	2328.461	2229.834	2452.769	3289.156232	2337.021197	2813.08871	0.49351649	0.110503
Csa2G011590	595.3253	566.7424	526.3592	462.9574	325.7576	369.8969	562.8089746	386.2039836	474.506479	0.543090634	0.116267
Csa1G042340	226.1395	187.5174	196.8204	126.9714	75.24543	138.7113	203.4924179	113.6427345	158.567576	0.844718948	0.133098
Csa3G872110	447.0199	547.8859	590.4613	728.6208	701.0672	773.9211	528.4556939	734.536355	631.496024	-0.473394177	0.107997
Csa3G435530	308.1808	236.7538	404.475	192.4106	188.1136	198.159	316.4698432	192.8943959	254.68212	0.716722142	0.129283
Csa7G430130	37.86521	33.52266	33.4053	59.57891	77.08068	86.9698	34.93105716	74.54313109	54.7370941	-1.09288554	0.132434
Csa2G408430	337.6315	430.5566	376.4868	494.2119	582.6933	539.4329	381.5582961	538.7793789	460.168838	-0.498204933	0.111368
Csa3G734090	651.0713	631.6926	604.004	468.8176	499.1892	453.564	628.9226117	473.8569524	551.389782	0.407122905	0.098485
Csa6G516990	745.7343	508.0778	538.9991	432.6796	330.3458	407.3269	597.6037176	390.1174404	493.860579	0.615504605	0.123262
Csa4G631560	112.5438	71.23565	84.86752	55.6721	33.95221	30.82474	89.54899852	40.14968029	64.8493394	1.154060825	0.130995
Csa6G291920	3274.289	3454.929	3434.426	2841.23	2710.671	2813.858	3387.881366	2788.586446	3088.23391	0.281012244	0.074657
Csa7G388300	591.118	544.7432	512.8165	838.9883	650.5977	792.6362	549.5592479	760.7406932	655.149971	-0.469110521	0.10821
Csa3G914030	320.8025	353.0355	315.0933	236.3622	242.2536	210.2688	329.6437438	229.6281882	279.635966	0.519366766	0.114586
Csa5G636490	455.4344	463.0317	415.3092	237.3389	369.8037	254.3041	444.5917407	287.148929	365.870335	0.628118818	0.124617
Csa6G013350	2353.954	2154.878	2552.346	1981.731	1672.834	1785.633	2353.725994	1813.39958	2083.56279	0.376623208	0.093845
Csa6G493900	237.7094	190.6601	310.579	152.3657	155.9966	137.6104	246.3161749	148.6576	197.486888	0.729748624	0.130267
Csa7G072880	3230.113	3620.447	3295.388	4756.546	3766.86	5048.652	3381.982619	4524.019094	3953.00086	-0.41954119	0.101071

Csa2G421020	369.1858	194.8504	233.8371	138.6919	155.079	157.4263	265.9577923	150.3990763	208.178434	0.82181903	0.132955
Csa1G002090	249.2793	214.7545	258.214	394.5882	547.8234	281.8262	240.7492643	408.0792667	324.414266	-0.762725402	0.131553
Csa5G633230	241.9166	197.9932	144.4554	122.0879	82.58645	117.7945	194.788396	107.4896386	151.139017	0.857928804	0.133483
Csa6G516960	772.0296	474.5551	997.6448	463.9341	476.2485	386.4101	748.0765164	442.1975878	595.137052	0.75877019	0.131452
Csa2G011420	569.03	400.1767	273.5623	235.3855	199.1251	268.6156	414.2563509	234.3754044	324.315878	0.821550991	0.133009
Csa2G223740	3444.682	3902.247	3696.252	3122.521	2539.074	2922.846	3681.060285	2861.480374	3271.27033	0.363562422	0.092312
Csa6G052710	997.1172	932.3489	901.9432	728.6208	730.4312	755.2061	943.8031049	738.086038	840.944571	0.354536682	0.090726
Csa6G366320	651.0713	642.1684	687.0658	896.6138	798.3356	873.0007	660.1018325	855.9833522	758.042592	-0.373980775	0.094282
Csa3G738980	596.3771	481.8882	617.5467	747.1781	805.6767	753.0044	565.2706513	768.6197133	666.945182	-0.442710677	0.105591
Csa6G290900	326.0615	339.4169	243.7684	209.0145	200.0427	186.0493	303.0822916	198.3688601	250.725576	0.608671682	0.123853
Csa6G502700	238.7612	228.3731	219.3916	152.3657	165.1729	139.8122	228.84196	152.4502796	190.64612	0.583257458	0.121793
Csa3G238150	92.55941	143.5189	91.18745	61.53232	59.64577	38.53092	109.0885769	53.23633572	81.1624563	1.028179563	0.132898
Csa2G382470	457.538	432.6518	687.9687	353.5666	313.8285	352.2827	526.0528095	339.8926252	432.972717	0.632294288	0.12569
Csa1G075600	3306.895	2961.517	3390.187	3982.997	3855.869	4118.405	3219.533061	3985.757128	3602.6451	-0.307693901	0.0819
Csa1G629040	6343.475	4289.853	7234.505	3991.787	3998.102	4325.372	5955.944129	4105.086679	5030.5154	0.537103196	0.118051
Csa5G603910	164.0826	138.281	163.4151	224.6418	223.901	251.0015	155.2595578	233.1814239	194.220491	-0.584617699	0.122503
Csa2G377370	1602.961	1051.773	1764.161	869.2661	1168.139	656.1266	1472.965034	897.8440159	1185.40453	0.713639719	0.130442
Csa2G416830	164.0826	151.8995	147.1639	91.81012	106.4448	88.07069	154.3820083	95.44185321	124.911931	0.690545524	0.129441
Csa1G595810	786.755	640.0732	673.5231	514.7227	544.1529	507.5073	700.1171058	522.1276482	611.122377	0.42236546	0.103633
Csa5G217680	1622.945	1668.8	1824.652	2076.472	2100.449	2154.429	1705.465539	2110.449742	1907.95764	-0.306808981	0.082625
Csa5G590070	1065.485	1104.153	930.8342	1307.806	1335.148	1311.152	1033.490584	1318.035252	1175.76292	-0.351756103	0.091651
Csa1G124000	1217.998	1156.532	1296.487	1635.002	1616.859	1424.543	1223.672064	1558.801318	1391.23669	-0.349299351	0.091218
Csa1G422470	120.9583	165.5181	251.894	58.60221	117.4563	83.66715	179.4568239	86.57521153	133.016018	1.052049741	0.132119
Csa3G016930	593.2217	508.0778	522.7478	751.0849	846.9699	640.7142	541.3490887	746.256351	643.80272	-0.464622509	0.110023
Csa1G045570	1223.257	1301.098	1206.202	1673.093	1418.652	1700.865	1243.519037	1597.536565	1420.5278	-0.361092926	0.093608

Csa5G434550	107.2848	73.33081	89.38175	121.1112	213.8071	159.6281	89.99911149	164.8488244	127.423968	-0.874663649	0.1338
Csa6G010030	2302.415	1547.28	2893.621	1560.772	1584.742	1115.195	2247.77228	1420.23642	1834.00435	0.662282977	0.128416
Csa5G160230	405.9992	425.3187	474.897	347.7064	280.7939	304.9447	435.4049768	311.1483613	373.276669	0.486063326	0.11312
Csa3G732440	219.8286	207.4214	214.8773	97.67034	131.2207	165.1325	214.042461	131.3411877	172.691824	0.707924211	0.130633
Csa1G599510	7157.577	7278.607	7356.389	9179.059	8132.012	9196.781	7264.191032	8835.950716	8050.07087	-0.282459418	0.077899
Csa4G364040	1669.225	2100.404	1905.005	2676.167	2299.574	2406.531	1891.544611	2460.757523	2176.15107	-0.379474005	0.097403
Csa3G319280	462.797	529.0294	505.5938	337.9394	388.1563	379.8048	499.1400781	368.6335035	433.886791	0.437476833	0.106796
Csa2G007410	975.0292	1083.201	985.9078	855.5922	769.8892	703.4646	1014.712637	776.3153338	895.513986	0.385490141	0.09858
Csa6G127300	1034.982	541.6004	1144.809	429.7495	510.2007	647.3195	907.1305408	529.0899176	718.110229	0.778951127	0.132843
Csa4G641610	3845.423	5003.257	3466.026	3220.191	2998.806	2795.143	4104.90169	3004.713394	3554.80754	0.449827337	0.108701
Csa6G499200	76.78224	39.80816	60.49068	15.62725	11.92915	34.12739	59.02702436	20.56126621	39.7941453	1.536619683	0.117884
Csa4G088750	745.7343	777.3066	966.9481	628.997	604.7163	613.1921	829.9963336	615.6351539	722.815744	0.432206711	0.106218

stat	pvalue	padj	annotation
27.49978	1.77E-166	3.25E-162	Auxin-responsive protein; contains IPR003311 (AUX/IAA protein)
25.54471	6.29E-144	5.78E-140	Indole-3-acetic acid-amido synthetase GH3.3, putative; contains IPR004993 (GH3 auxin-responsive promoter)
22.47519	7.26E-112	4.45E-108	Indole-3-acetic acid-amido synthetase GH3.3, putative; contains IPR004993 (GH3 auxin-responsive promoter)
20.32172	8.26E-92	3.80E-88	Probable membrane-associated kinase regulator 6
19.80457	2.72E-87	1.00E-83	Auxin-responsive protein; contains IPR003311 (AUX/IAA protein)
18.2522	1.99E-74	6.09E-71	Plant-specific domain TIGR01570 family protein; contains IPR006460 (Protein of unknown function DUF617, plant)
17.64216	1.17E-69	3.07E-66	Sodium/hydrogen exchanger; contains IPR006153 (Cation/H+ exchanger), IPR018422 (Cation/H+ exchanger, CPA1 family)
17.55106	5.84E-69	1.34E-65	Indole-3-acetic acid-amido synthetase GH3.3, putative; contains IPR004993 (GH3 auxin-responsive promoter)
16.07003	4.14E-58	8.46E-55	Putative jasmonate O-methyltransferase; contains IPR005299 (SAM dependent carboxyl methyltransferase)
15.63945	3.92E-55	7.21E-52	Unknown protein
15.58143	9.74E-55	1.63E-51	Glutaredoxin; contains IPR011905 (Glutaredoxin-like, plant II), IPR012336 (Thioredoxin-like fold)
15.41239	1.35E-53	2.07E-50	CM0216.330.nc protein; contains IPR004320 (Protein of unknown function DUF241, plant)
15.37075	2.57E-53	3.64E-50	Dof zinc finger protein; contains IPR003851 (Zinc finger, Dof-type)
15.19292	3.94E-52	5.17E-49	Auxin-responsive protein; contains IPR003311 (AUX/IAA protein)
14.92275	2.34E-50	2.87E-47	Auxin-responsive protein; contains IPR003311 (AUX/IAA protein)
14.90825	2.91E-50	3.35E-47	Unknown protein
14.87883	4.52E-50	4.89E-47	Auxin efflux carrier; contains IPR004776 (Auxin efflux carrier)
13.88523	7.78E-44	7.95E-41	Plant-specific domain TIGR01570 family protein; contains IPR006460 (Protein of unknown function DUF617, plant)
13.34313	1.30E-40	1.26E-37	S-adenosylmethionine decarboxylase proenzyme; contains IPR001985 (S-adenosylmethionine decarboxylase), IPR016067 (S-adenosylmethionine decarboxylase, core), IPR018167 (S-adenosylmethionine decarboxylase subgroup)
12.87649	6.10E-38	5.61E-35	BTB/POZ domain-containing protein; contains IPR011333 (BTB/POZ fold), IPR027356 (NPH3 domain)
12.8538	8.19E-38	7.17E-35	BTB/POZ domain-containing protein; contains IPR011333 (BTB/POZ fold), IPR027356 (NPH3 domain)
12.46406	1.17E-35	9.80E-33	Putative HLH DNA-binding domain superfamily protein; contains IPR011598 (Myc-type, basic helix-loop-helix (bHLH) domain), IPR025610 (Transcription factor MYC/MYB N-terminal)
12.44725	1.45E-35	1.16E-32	Auxin-responsive protein; contains IPR003311 (AUX/IAA protein)
12.35213	4.74E-35	3.63E-32	NAD(P)H-quinone oxidoreductase subunit 6; contains IPR009902 (Protein of unknown function DUF1442)
12.11982	8.29E-34	6.10E-31	Glutaredoxin-C9; contains IPR011905 (Glutaredoxin-like, plant II), IPR012336 (Thioredoxin-like fold)
11.88879	1.35E-32	9.57E-30	Short-chain dehydrogenase/reductase family protein; contains IPR002347 (Glucose/ribitol dehydrogenase)
11.78688	4.56E-32	3.11E-29	Phosphatidylinositol-4-phosphate 5-kinase, putative; contains IPR023610 (Phosphatidylinositol-4-phosphate 5-kinase), IPR027483 (Phosphatidylinositol-4-phosphate 5-kinase, C-terminal), IPR027484 (Phosphatidylinositol-4-phosphate 5-kinase, N-terminal domain)

11.38002	5.26E-30	3.45E-27	Putative carboxyl-terminal proteinase; contains IPR004314 (Domain of unknown function DUF239), IPR025521 (Domain of unknown function DUF4409)
11.18349	4.91E-29	3.12E-26	Homeobox-leucine zipper protein; contains IPR003106 (Leucine zipper, homeobox-associated), IPR006712 (HD-ZIP protein, N-terminal), IPR009057 (Homeodomain-like)
10.94469	7.05E-28	4.32E-25	Auxin-responsive protein; contains IPR003311 (AUX/IAA protein)
10.77804	4.37E-27	2.59E-24	Auxin responsive protein; contains IPR003311 (AUX/IAA protein)
10.65671	1.62E-26	9.32E-24	Unknown protein
10.51421	7.43E-26	4.14E-23	DNA binding protein, putative; contains IPR011598 (Myc-type, basic helix-loop-helix (bHLH) domain)
10.4973	8.89E-26	4.81E-23	Receptor-like protein kinase; contains IPR001611 (Leucine-rich repeat), IPR003591 (Leucine-rich repeat, typical subtype), IPR011009 (Protein kinase-like domain), IPR013210 (Leucine-rich repeat-containing N-terminal, type 2)
10.23682	1.36E-24	7.13E-22	Auxin responsive protein; contains IPR003311 (AUX/IAA protein)
10.20943	1.80E-24	9.19E-22	F2E2.8
10.0664	7.78E-24	3.87E-21	Unknown protein
9.965638	2.15E-23	1.04E-20	Auxin-responsive protein; contains IPR003311 (AUX/IAA protein)
9.948027	2.57E-23	1.21E-20	At5g51670-like protein; contains IPR007700 (Protein of unknown function DUF668), IPR021864 (Protein of unknown function DUF3475)
9.928347	3.13E-23	1.44E-20	6-phosphogluconolactonase, putative; contains IPR006148 (Glucosamine/galactosamine-6-phosphate isomerase)
-9.90301	4.04E-23	1.81E-20	Monooxygenase, putative; contains IPR013027 (FAD-dependent pyridine nucleotide-disulphide oxidoreductase), IPR020946 (Flavin monooxygenase-like)
9.784021	1.32E-22	5.77E-20	Auxin efflux carrier; contains IPR004776 (Auxin efflux carrier)
9.644015	5.21E-22	2.23E-19	F16L1.4 protein
9.61264	7.07E-22	2.96E-19	Metal ion binding protein; contains IPR006121 (Heavy metal-associated domain, HMA)
9.24782	2.29E-20	9.36E-18	Auxin-responsive protein; contains IPR003311 (AUX/IAA protein)
9.112947	8.02E-20	3.21E-17	Pre-mRNA-splicing factor CWC22 homolog
9.020624	1.87E-19	7.32E-17	Auxin response factor, putative; contains IPR003311 (AUX/IAA protein), IPR010525 (Auxin response factor), IPR015300 (DNA-binding pseudobarrel domain)
8.894158	5.89E-19	2.26E-16	Transcription factor, putative; contains IPR011598 (Myc-type, basic helix-loop-helix (bHLH) domain)
8.570547	1.03E-17	3.87E-15	Auxin transporter-like protein 3; contains IPR013057 (Amino acid transporter, transmembrane)
8.522646	1.56E-17	5.74E-15	Unknown protein
8.309185	9.64E-17	3.47E-14	Serine/threonine-protein kinase; contains IPR011009 (Protein kinase-like domain)
8.300869	1.03E-16	3.66E-14	Putative HLH DNA-binding domain superfamily protein; contains IPR011598 (Myc-type, basic helix-loop-helix (bHLH) domain), IPR025610 (Transcription factor MYC/MYB N-terminal)
8.268198	1.36E-16	4.72E-14	Dof zinc finger protein; contains IPR003851 (Zinc finger, Dof-type)
8.219565	2.04E-16	6.96E-14	Fas-associated factor 1-like protein; contains IPR001012 (UBX), IPR006577 (UAS), IPR012336 (Thioredoxin-like fold)

8.118455	4.72E-16	1.58E-13	Putative serine rich protein
8.093018	5.82E-16	1.91E-13	Putative serine rich protein
7.921876	2.34E-15	7.55E-13	Unknown protein
7.801699	6.11E-15	1.94E-12	WUSCHEL-related homeobox 3B; contains IPR009057 (Homeodomain-like)
7.666669	1.77E-14	5.50E-12	TPD1
7.663966	1.80E-14	5.53E-12	Auxin-responsive protein; contains IPR003311 (AUX/IAA protein)
7.548339	4.41E-14	1.33E-11	Protein phosphatase 2c, putative; contains IPR001932 (Protein phosphatase 2C (PP2C)-like), IPR015655 (Protein phosphatase 2C)
7.505201	6.13E-14	1.82E-11	WUSCHEL-related homeobox 14; contains IPR009057 (Homeodomain-like)
7.40971	1.27E-13	3.69E-11	Auxin-responsive protein; contains IPR003311 (AUX/IAA protein)
7.399126	1.37E-13	3.94E-11	Receptor-like protein kinase; contains IPR024788 (Malectin-like carbohydrate-binding domain)
7.37606	1.63E-13	4.61E-11	F14D16.16
7.328883	2.32E-13	6.47E-11	Cytochrome P450, putative; contains IPR001128 (Cytochrome P450)
7.310822	2.66E-13	7.29E-11	Structural constituent of cell wall, putative; contains IPR008889 (VQ)
7.22592	4.98E-13	1.35E-10	Receptor-like kinase; contains IPR001611 (Leucine-rich repeat), IPR011009 (Protein kinase-like domain), IPR013210 (Leucine-rich repeat-containing N-terminal, type 2), IPR013320 (Concanavalin A-like lectin/glucanase, subgroup)
7.212898	5.48E-13	1.46E-10	Acetyl esterase; contains IPR013094 (Alpha/beta hydrolase fold-3)
7.206503	5.74E-13	1.51E-10	S-adenosylmethionine decarboxylase proenzyme; contains IPR001985 (S-adenosylmethionine decarboxylase), IPR016067 (S-adenosylmethionine decarboxylase, core), IPR018167 (S-adenosylmethionine decarboxylase subgroup)
7.185835	6.68E-13	1.72E-10	Serine/threonine-protein kinase; contains IPR000858 (S-locus glycoprotein), IPR001480 (Bulb-type lectin domain)
7.18473	6.73E-13	1.72E-10	NAC domain-containing protein, putative; contains IPR003441 (No apical meristem (NAM) protein)
6.989903	2.75E-12	6.93E-10	Protein Brevis radix-like 4; contains IPR013591 (Brevis radix-like domain)
6.909806	4.85E-12	1.21E-09	Class III homeobox-leucine zipper protein; contains IPR002913 (START domain), IPR009057 (Homeodomain-like), IPR013978 (MEKHLA), IPR023393 (START-like domain)
6.864478	6.67E-12	1.64E-09	Putative DUF566 domain containing family protein; contains IPR007573 (Protein of unknown function DUF566)
6.845569	7.62E-12	1.84E-09	T10O24.15, related; contains IPR025322 (Protein of unknown function DUF4228)
6.810254	9.74E-12	2.33E-09	Unknown protein
6.707366	1.98E-11	4.67E-09	Transcription factor, putative; contains IPR005333 (Transcription factor, TCP)
6.672333	2.52E-11	5.86E-09	Unknown protein
6.647425	2.98E-11	6.86E-09	Cytochrome P450, putative; contains IPR001128 (Cytochrome P450)
6.634263	3.26E-11	7.40E-09	Multidrug resistance protein MdtK; contains IPR002528 (Multi antimicrobial extrusion protein)
6.578127	4.76E-11	1.07E-08	Heat shock protein-related protein; contains IPR004176 (Clp, N-terminal), IPR023150 (Double Clp-N motif)
6.55068	5.73E-11	1.27E-08	AT5g57340/MJB24_15
6.546318	5.90E-11	1.29E-08	Auxin-responsive protein; contains IPR003311 (AUX/IAA protein)

6.496663	8.21E-11	1.78E-08	Protein BREVIS RADIX; contains IPR013591 (Brevis radix-like domain)
6.485692	8.83E-11	1.89E-08	Abhydrolase domain-containing protein FAM108C1
6.476884	9.36E-11	1.98E-08	Growth-regulating factor; contains IPR014977 (WRC), IPR014978 (Glutamine-Leucine-Glutamine, QLQ)
6.445156	1.15E-10	2.41E-08	Receptor-like protein kinase; contains IPR001611 (Leucine-rich repeat), IPR003591 (Leucine-rich repeat, typical subtype), IPR011009 (Protein kinase-like domain)
-6.38844	1.68E-10	3.46E-08	Pleiotropic drug resistance ABC transporter family protein; contains IPR013525 (ABC-2 type transporter), IPR013581 (Plant PDR ABC transporter associated), IPR027417 (P-loop containing nucleoside triphosphate hydrolase)
6.381948	1.75E-10	3.57E-08	Ethylene-responsive transcription factor; contains IPR016177 (DNA-binding, integrase-type)
6.325715	2.52E-10	5.09E-08	Protein kinase; contains IPR011009 (Protein kinase-like domain)
6.322126	2.58E-10	5.16E-08	Coilin
6.292187	3.13E-10	6.19E-08	DNA-binding protein-like; contains IPR011598 (Myc-type, basic helix-loop-helix (bHLH) domain)
-6.28272	3.33E-10	6.51E-08	Ice binding protein, putative; contains IPR001849 (Pleckstrin homology domain), IPR008546 (Domain of unknown function DUF828), IPR013666 (Pleckstrin-like, plant)
6.260229	3.84E-10	7.43E-08	Transcription factor bHLH106; contains IPR011598 (Myc-type, basic helix-loop-helix (bHLH) domain)
6.258889	3.88E-10	7.43E-08	BTB/POZ domain-containing protein; contains IPR011333 (BTB/POZ fold), IPR027356 (NPH3 domain)
-6.20113	5.61E-10	1.06E-07	Protein FANTASTIC FOUR 3; contains IPR021410 (Protein of unknown function DUF3049)
6.197197	5.75E-10	1.08E-07	C2 domain-containing protein; contains IPR008973 (C2 calcium/lipid-binding domain, CaLB)
6.168194	6.91E-10	1.28E-07	Ethylene-responsive transcription factor; contains IPR016177 (DNA-binding, integrase-type)
6.134852	8.52E-10	1.57E-07	Cellulose synthase; contains IPR005150 (Cellulose synthase), IPR013083 (Zinc finger, RING/FYVE/PHD-type)
-6.12237	9.22E-10	1.68E-07	9-cis-epoxycarotenoid dioxygenase, putative; contains IPR004294 (Carotenoid oxygenase)
6.095637	1.09E-09	1.97E-07	WAT1-like protein; contains IPR000620 (Drug/metabolite transporter)
6.090224	1.13E-09	2.01E-07	Dof zinc finger protein; contains IPR003851 (Zinc finger, Dof-type)
6.059521	1.37E-09	2.41E-07	NAC domain-containing protein, putative; contains IPR003441 (No apical meristem (NAM) protein)
6.048473	1.46E-09	2.56E-07	G protein gamma subunit 1; contains IPR015898 (G-protein gamma-like domain)
6.032853	1.61E-09	2.79E-07	Serine/threonine protein kinase, putative; contains IPR011009 (Protein kinase-like domain)
5.975732	2.29E-09	3.94E-07	Unknown protein
-5.93358	2.96E-09	5.05E-07	1-aminocyclopropane-1-carboxylate oxidase-like protein; contains IPR005123 (Oxoglutarate/iron-dependent dioxygenase), IPR026992 (Non-haem dioxygenase N-terminal domain), IPR027443 (Isopenicillin N synthase-like)
5.907088	3.48E-09	5.87E-07	Calmodulin, putative; contains IPR011992 (EF-hand-like domain)
5.813605	6.11E-09	1.02E-06	RING finger family protein; contains IPR013083 (Zinc finger, RING/FYVE/PHD-type)
5.809097	6.28E-09	1.04E-06	Heavy-metal-associated domain-containing protein, putative, expressed; contains IPR006121 (Heavy metal-associated domain, HMA)
5.76084	8.37E-09	1.37E-06	WUSCHEL-related homeobox; contains IPR009057 (Homeodomain-like)
5.723798	1.04E-08	1.70E-06	Transcription factor, putative; contains IPR025422 (DOG1 domain)

5.714915	1.10E-08	1.77E-06	Putative two-component response regulator family protein; contains IPR009057 (Homeodomain-like)
5.69859	1.21E-08	1.93E-06	Zinc finger-homeodomain protein 3; contains IPR006456 (ZF-HD homeobox protein, Cys/His-rich dimerisation domain), IPR009057 (Homeodomain-like)
5.684957	1.31E-08	2.06E-06	Multidrug resistance protein MdtK; contains IPR002528 (Multi antimicrobial extrusion protein)
-5.68606	1.30E-08	2.06E-06	EPIDERMAL PATTERNING FACTOR-like protein
5.665224	1.47E-08	2.27E-06	U-box domain-containing protein; contains IPR013083 (Zinc finger, RING/FYVE/PHD-type), IPR016024 (Armadillo-type
5.66586	1.46E-08	2.27E-06	Abhydrolase domain-containing protein FAM108C1
5.650612	1.60E-08	2.45E-06	Auxin-responsive protein; contains IPR003311 (AUX/IAA protein)
5.609415	2.03E-08	3.09E-06	BTB/POZ domain-containing protein; contains IPR011333 (BTB/POZ fold)
5.557978	2.73E-08	4.11E-06	Tetraspanin-3; contains IPR018499 (Tetraspanin/Peripherin)
-5.55359	2.80E-08	4.18E-06	Lipid transfer protein; contains IPR016140 (Bifunctional inhibitor/plant lipid transfer protein/seed storage helical domain)
5.552528	2.82E-08	4.18E-06	Rho GDP-dissociation inhibitor, putative; contains IPR000406 (RHO protein GDP dissociation inhibitor), IPR014756 (Immunoglobulin E-set)
-5.54747	2.90E-08	4.26E-06	Bzip-like transcription factor-like; contains IPR006867 (Domain of unknown function DUF632), IPR006868 (Domain of unknown function DUF630)
5.540466	3.02E-08	4.40E-06	Serine/threonine protein kinase, putative; contains IPR011009 (Protein kinase-like domain)
-5.48526	4.13E-08	5.98E-06	Formin-like protein; contains IPR003104 (Actin-binding FH2/DRF autoregulatory)
5.475553	4.36E-08	6.22E-06	Auxin-responsive protein; contains IPR003311 (AUX/IAA protein)
5.476774	4.33E-08	6.22E-06	AT-hook DNA-binding protein; contains IPR014476 (Predicted AT-hook DNA-binding)
5.468336	4.54E-08	6.43E-06	F-box protein; contains IPR001810 (F-box domain, cyclin-like), IPR002893 (Zinc finger, MYND-type), IPR011990 (Tetratricopeptide-like helical)
5.45997	4.76E-08	6.69E-06	Transcription factor AIG1; contains IPR011598 (Myc-type, basic helix-loop-helix (bHLH) domain)
5.449408	5.05E-08	7.04E-06	Auxin transporter-like protein; contains IPR013057 (Amino acid transporter, transmembrane)
5.445898	5.15E-08	7.13E-06	Glutamate receptor 1.2; contains IPR001320 (Ionotropic glutamate receptor), IPR001638 (Extracellular solute-binding protein, family 3)
-5.41879	6.00E-08	8.23E-06	CBL-interacting protein kinase 9; contains IPR020636 (Calcium/calmodulin-dependent/calcium-dependent protein kinase)
5.332158	9.71E-08	1.32E-05	Multidrug resistance protein MdtK; contains IPR002528 (Multi antimicrobial extrusion protein)
5.31609	1.06E-07	1.43E-05	F-box family protein; contains IPR001810 (F-box domain, cyclin-like)
5.296686	1.18E-07	1.58E-05	Cytochrome P450, putative; contains IPR001128 (Cytochrome P450)
5.256408	1.47E-07	1.96E-05	C4-dicarboxylate transporter/malic acid transport family protein; contains IPR004695 (C4-dicarboxylate transporter/malic acid transport protein)
5.248306	1.54E-07	2.03E-05	Probable peptide/nitrate transporter; contains IPR000109 (Proton-dependent oligopeptide transporter family), IPR016196 (Major facilitator superfamily domain, general substrate transporter)
5.24709	1.55E-07	2.03E-05	Putative uncharacterized protein OJ1004_D04.13

5.211711	1.87E-07	2.44E-05	Putative carboxyl-terminal proteinase; contains IPR004314 (Domain of unknown function DUF239), IPR025521 (Domain of unknown function DUF4409)
5.187454	2.13E-07	2.76E-05	18.2 kDa class I heat shock protein; contains IPR008978 (HSP20-like chaperone)
5.177168	2.25E-07	2.90E-05	Pentapeptide repeat protein (Precursor); contains IPR001646 (Pentapeptide repeat)
5.150437	2.60E-07	3.32E-05	Thylakoid lumenal 17.9 kDa protein
5.144252	2.69E-07	3.41E-05	Putative MYB transcription factor; contains IPR009057 (Homeodomain-like)
-5.14039	2.74E-07	3.45E-05	Probable membrane-associated kinase regulator 5
-5.09566	3.48E-07	4.35E-05	ABC transporter G family member; contains IPR013525 (ABC-2 type transporter), IPR027417 (P-loop containing nucleoside triphosphate hydrolase)
-5.08954	3.59E-07	4.46E-05	Cytokinin oxidase/dehydrogenase 1; contains IPR016164 (FAD-linked oxidase-like, C-terminal), IPR016166 (FAD-binding, type 2), IPR016170 (Vanillyl-alcohol oxidase/Cytokinin dehydrogenase C-terminal domain)
-5.08008	3.77E-07	4.66E-05	Hfr-2-like protein; contains IPR005830 (Aerolysin/haemolysin/leukocidin toxin), IPR008998 (Agglutinin), IPR023307 (Aerolysin-like toxin, beta complex domain)
5.07503	3.87E-07	4.75E-05	Membrane protein-like; contains IPR002781 (Transmembrane protein TauE like)
5.069102	4.00E-07	4.87E-05	Aspartic proteinase nepenthesin-1, putative; contains IPR001461 (Peptidase A1), IPR021109 (Aspartic peptidase)
5.063861	4.11E-07	4.97E-05	Cytokinin riboside 5'-monophosphate phosphoribohydrolase-like; contains IPR005269 (Cytokinin riboside 5'-monophosphate phosphoribohydrolase LOG)
4.988858	6.07E-07	7.30E-05	Putative uncharacterized protein At2g28140/F24D13.7; contains IPR012862 (Protein of unknown function DUF1635)
4.980162	6.35E-07	7.59E-05	Catalytic/ hydrolase; contains IPR010237 (Pyrimidine 5-nucleotidase), IPR023214 (HAD-like domain)
-4.97582	6.50E-07	7.71E-05	Receptor protein kinase-like protein; contains IPR001611 (Leucine-rich repeat), IPR003591 (Leucine-rich repeat, typical subtype), IPR011009 (Protein kinase-like domain)
-4.97412	6.55E-07	7.73E-05	Probable peptide/nitrate transporter; contains IPR000109 (Proton-dependent oligopeptide transporter family), IPR016196 (Major facilitator superfamily domain, general substrate transporter)
4.970857	6.67E-07	7.81E-05	Myb family transcription factor; contains IPR009057 (Homeodomain-like)
-4.96113	7.01E-07	8.16E-05	Ethphon-induced protein; contains IPR007650 (Protein of unknown function DUF581)
4.959211	7.08E-07	8.19E-05	Homeobox-leucine zipper protein; contains IPR009057 (Homeodomain-like)
4.957481	7.14E-07	8.21E-05	NAC domain-containing protein, putative; contains IPR003441 (No apical meristem (NAM) protein)
4.954977	7.23E-07	8.26E-05	Histone acetyltransferase
-4.93509	8.01E-07	9.09E-05	Hfr-2-like protein; contains IPR005830 (Aerolysin/haemolysin/leukocidin toxin), IPR008998 (Agglutinin), IPR023307 (Aerolysin-like toxin, beta complex domain)
-4.92079	8.62E-07	9.72E-05	Putative cytochrome P450; contains IPR001128 (Cytochrome P450)
4.914858	8.88E-07	9.96E-05	mRNA, 952 bp sequence; contains IPR025322 (Protein of unknown function DUF4228)
4.907813	9.21E-07	0.000103	Emb|CAB83157.1
4.896687	9.75E-07	0.000108	WD-repeat protein, putative; contains IPR015943 (WD40/YVTN repeat-like-containing domain)

4.870994	1.11E-06	0.000122	WD-repeat protein, putative; contains IPR015943 (WD40/YVTN repeat-like-containing domain)
4.863118	1.16E-06	0.000126	Ethylene-responsive transcription factor 7; contains IPR016177 (DNA-binding, integrase-type)
-4.85872	1.18E-06	0.000129	Cytochrome P450; contains IPR001128 (Cytochrome P450)
4.850309	1.23E-06	0.000133	Aspartyl protease-like protein; contains IPR001461 (Peptidase A1), IPR021109 (Aspartic peptidase)
-4.85131	1.23E-06	0.000133	U-box domain-containing protein; contains IPR016024 (Armadillo-type fold)
-4.8331	1.34E-06	0.000144	Cytochrome P450, putative; contains IPR001128 (Cytochrome P450)
4.798252	1.60E-06	0.00017	Boron transporter, putative; contains IPR003020 (Bicarbonate transporter, eukaryotic)
-4.78983	1.67E-06	0.000176	Calmodulin binding protein, putative; contains IPR000048 (IQ motif, EF-hand binding site), IPR025064 (Domain of unknown function DUF4005)
4.776127	1.79E-06	0.000188	Chaperone protein dnaJ; contains IPR001623 (DnaJ domain)
4.773989	1.81E-06	0.000189	Putative RNA-binding protein; contains IPR012677 (Nucleotide-binding, alpha-beta plait)
4.755056	1.98E-06	0.000206	NHL1; contains IPR004864 (Late embryogenesis abundant protein, LEA-14)
4.738738	2.15E-06	0.000222	Putative uncharacterized protein T21H19_30
4.697628	2.63E-06	0.00027	Auxin transporter-like protein 3; contains IPR013057 (Amino acid transporter, transmembrane)
4.692518	2.70E-06	0.000276	Hexose transporter; contains IPR005828 (General substrate transporter), IPR016196 (Major facilitator superfamily domain, general substrate transporter)
4.679567	2.87E-06	0.000292	Putative DUF566 domain containing family protein; contains IPR007573 (Protein of unknown function DUF566)
4.670048	3.01E-06	0.000304	Zinc finger family protein; contains IPR011016 (Zinc finger, RING-CH-type), IPR013083 (Zinc finger, RING/FYVE/PHD-type)
4.645258	3.40E-06	0.000341	CM0216.240.nc protein; contains IPR004320 (Protein of unknown function DUF241, plant)
4.642332	3.44E-06	0.000344	Male sterility MS5 family protein; contains IPR011990 (Tetratricopeptide-like helical)
-4.63814	3.52E-06	0.000348	Desumoylating isopeptidase 2; contains IPR008580 (Domain of unknown function DUF862, eukaryotic)
-4.63824	3.51E-06	0.000348	Amine oxidase; contains IPR000269 (Copper amine oxidase)
4.63475	3.57E-06	0.000351	Patatin T5, putative; contains IPR016035 (Acyl transferase/acyl hydrolase/lysophospholipase)
4.619484	3.85E-06	0.000376	[Protein-PII] uridylyltransferase; contains IPR002912 (ACT domain)
4.615483	3.92E-06	0.000382	Ethylene-responsive transcription factor 7; contains IPR016177 (DNA-binding, integrase-type)
4.612923	3.97E-06	0.000384	Cytochrome P450; contains IPR001128 (Cytochrome P450)
4.591418	4.40E-06	0.000424	Receptor-like protein kinase 2.33; contains IPR001611 (Leucine-rich repeat)
4.583675	4.57E-06	0.000438	GDSL esterase/lipase; contains IPR001087 (Lipase, GDSL)
4.581533	4.62E-06	0.00044	Auxin response factor 1; contains IPR003311 (AUX/IAA protein), IPR010525 (Auxin response factor), IPR015300 (DNA-binding pseudobarrel domain)
4.570329	4.87E-06	0.000462	Unknown protein
4.565896	4.97E-06	0.000469	Leucine-rich repeat receptor-like serine/threonine-protein kinase
4.558984	5.14E-06	0.000482	Gb|AAF17687.1; contains IPR007650 (Protein of unknown function DUF581)
4.557474	5.18E-06	0.000483	Phosphate-responsive 1 family protein; contains IPR006766 (Phosphate-induced protein 1)

4.543718	5.53E-06	0.000513	Cyclin d, putative; contains IPR013763 (Cyclin-like)
-4.5372	5.70E-06	0.000527	Nitrate transporter; contains IPR000109 (Proton-dependent oligopeptide transporter family), IPR016196 (Major facilitator superfamily domain, general substrate transporter)
-4.53375	5.79E-06	0.000533	Sulfate transporter; contains IPR001902 (Sulphate anion transporter)
4.530776	5.88E-06	0.000535	Unknown protein
4.531616	5.85E-06	0.000535	Putative carboxyl-terminal proteinase; contains IPR004314 (Domain of unknown function DUF239), IPR025521 (Domain of unknown function DUF4409)
4.509256	6.51E-06	0.000589	Ethylene-responsive transcription factor 6; contains IPR016177 (DNA-binding, integrase-type)
4.501325	6.75E-06	0.000609	Fasciclin-like arabinogalactan protein 12.2; contains IPR000782 (FAS1 domain)
4.497947	6.86E-06	0.000616	Stress responsive A/B barrel domain family protein; contains IPR011008 (Dimeric alpha-beta barrel)
-4.48534	7.28E-06	0.00065	UDP-glycosyltransferase 1; contains IPR002213 (UDP-glucuronosyl/UDP-glucosyltransferase)
4.4809	7.43E-06	0.00066	Kelch domain-containing protein 3; contains IPR011043 (Galactose oxidase/kelch, beta-propeller)
-4.47995	7.47E-06	0.00066	Cytochrome P450; contains IPR001128 (Cytochrome P450)
4.4668	7.94E-06	0.000699	Spermidine synthase; contains IPR001045 (Spermidine/spermine synthases family)
-4.45999	8.20E-06	0.000718	Two-component response regulator-like protein; contains IPR009057 (Homeodomain-like), IPR011006 (CheY-like
-4.44963	8.60E-06	0.00075	Unknown protein
4.445899	8.75E-06	0.000759	Chaperone protein dnaJ; contains IPR001623 (DnaJ domain)
-4.43364	9.27E-06	0.0008	F-box family protein; contains IPR001810 (F-box domain, cyclin-like), IPR006553 (Leucine-rich repeat, cysteine-containing subtype)
4.422228	9.77E-06	0.000839	Phosphoadenosine phosphosulfate reductase; contains IPR004508 (Thioredoxin-independent 5'-adenylylsulphate reductase), IPR012336 (Thioredoxin-like fold)
4.417655	9.98E-06	0.000853	Unknown protein
4.41522	1.01E-05	0.000859	Probable receptor-like protein kinase; contains IPR011009 (Protein kinase-like domain), IPR013320 (Concanavalin A-like lectin/glucanase, subgroup)
4.383913	1.17E-05	0.000988	DNA-directed RNA polymerase II subunit RPB7; contains IPR005576 (RNA polymerase Rpb7, N-terminal), IPR012340 (Nucleic acid-binding, OB-fold)
4.375282	1.21E-05	0.001023	Unknown protein
-4.36279	1.28E-05	0.001078	Kinase, PfkB family; contains IPR011611 (Carbohydrate kinase PfkB)
4.360864	1.30E-05	0.001083	WUSCHEL-related homeobox; contains IPR009057 (Homeodomain-like)
4.344713	1.39E-05	0.00116	Ethylene-responsive transcription factor 7; contains IPR016177 (DNA-binding, integrase-type)
-4.32268	1.54E-05	0.001277	Ethylene receptor; contains IPR006458 (Ovate protein family, C-terminal)
4.315084	1.60E-05	0.001316	DNA-binding protein-like; contains IPR011598 (Myc-type, basic helix-loop-helix (bHLH) domain)
4.306699	1.66E-05	0.00136	NAC domain protein,; contains IPR003441 (No apical meristem (NAM) protein)
4.304132	1.68E-05	0.00137	Germin-like protein 5; contains IPR001929 (Germin)

-4.29356	1.76E-05	0.001431	Transcription factor, putative; contains IPR006869 (Domain of unknown function DUF547), IPR025757 (Ternary complex factor MIP1, leucine-zipper)
4.275601	1.91E-05	0.001544	Chaperone protein dnaj 11; contains IPR001623 (DnaJ domain)
4.268014	1.97E-05	0.001591	Glutamate decarboxylase
-4.26697	1.98E-05	0.001591	Putative E3 ubiquitin-protein ligase LIN-2; contains IPR016024 (Armadillo-type fold)
-4.26421	2.01E-05	0.001604	HAD-superfamily hydrolase, subfamily IA, variant 3; contains IPR006402 (HAD-superfamily hydrolase, subfamily IA, variant 3), IPR023214 (HAD-like domain)
4.259444	2.05E-05	0.001632	UDP-glycosyltransferase 1; contains IPR002213 (UDP-glucuronosyl/UDP-glucosyltransferase)
4.233364	2.30E-05	0.001825	Phosphatidylserine synthase, putative; contains IPR004277 (Phosphatidyl serine synthase)
4.221292	2.43E-05	0.001917	Peptide methionine sulfoxide reductase MsrA; contains IPR002569 (Peptide methionine sulphoxide reductase MsrA)
4.216861	2.48E-05	0.001947	Unknown protein
4.205458	2.61E-05	0.002039	Nucleic acid binding protein, putative; contains IPR007087 (Zinc finger, C2H2), IPR012317 (Poly(ADP-ribose) polymerase, catalytic domain)
4.187513	2.82E-05	0.002198	Histidine phosphotransfer protein; contains IPR008207 (Signal transduction histidine kinase, phosphotransfer (Hpt)
4.186013	2.84E-05	0.002203	Peroxidase; contains IPR010255 (Haem peroxidase)
4.182664	2.88E-05	0.002217	Unknown protein
4.183423	2.87E-05	0.002217	Expansin; contains IPR007118 (Expansin/Lol pI), IPR014733 (Barwin-like endoglucanase)
4.172895	3.01E-05	0.002305	Unknown protein
4.170714	3.04E-05	0.002317	Phytochrome-interacting factor 3; contains IPR011598 (Myc-type, basic helix-loop-helix (bHLH) domain)
4.1698	3.05E-05	0.002317	OTU-like cysteine protease family protein expressed; contains IPR003323 (Ovarian tumour, otubain)
-4.15731	3.22E-05	0.002437	Ubiquitin ligase protein; contains IPR013083 (Zinc finger, RING/FYVE/PHD-type)
4.13473	3.55E-05	0.002678	[Protein-PII] uridylyltransferase; contains IPR002912 (ACT domain)
4.125075	3.71E-05	0.002759	Anthocyanin 5-aromatic acyltransferase, putative; contains IPR003480 (Transferase), IPR023213 (Chloramphenicol acetyltransferase-like domain)
4.125986	3.69E-05	0.002759	Reductase 2; contains IPR001395 (Aldo/keto reductase), IPR023210 (NADP-dependent oxidoreductase domain)
4.126804	3.68E-05	0.002759	Gb|AAF04428.1
4.120993	3.77E-05	0.002797	Putative auxin/aluminum-responsive protein; contains IPR024286 (Domain of unknown function DUF3700)
4.118014	3.82E-05	0.002822	Auxin transporter-like protein; contains IPR013057 (Amino acid transporter, transmembrane)
4.116802	3.84E-05	0.002826	Cytokinin riboside 5'-monophosphate phosphoribohydrolase-like; contains IPR005269 (Cytokinin riboside 5'-monophosphate phosphoribohydrolase LOG)
4.115591	3.86E-05	0.002829	Putative uncharacterized protein OSJNBa0005K07.5
4.109073	3.97E-05	0.002899	F-box family protein; contains IPR001810 (F-box domain, cyclin-like)
4.102704	4.08E-05	0.002968	Flavin-containing monooxygenase family protein; contains IPR020946 (Flavin monooxygenase-like)
4.073408	4.63E-05	0.003354	GATA transcription factor; contains IPR016679 (Transcription factor, GATA, plant)

4.071987	4.66E-05	0.003362	Phi-1-like phosphate-induced protein; contains IPR006766 (Phosphate-induced protein 1)
4.06189	4.87E-05	0.003497	Low psii accumulation2 protein
4.05766	4.96E-05	0.003547	EPIDERMAL PATTERNING FACTOR-like protein
4.037579	5.40E-05	0.003849	Xyloglucan endotransglucosylase/hydrolase 13; contains IPR008985 (Concanavalin A-like lectin/glucanases superfamily), IPR016455 (Xyloglucan endotransglucosylase/hydrolase)
4.01031	6.06E-05	0.004306	Late embryogenesis abundant protein 3L-1; contains IPR004926 (Late embryogenesis abundant protein, LEA-5)
4.006253	6.17E-05	0.004363	PLATZ transcription factor; contains IPR006734 (Protein of unknown function DUF597)
4.001272	6.30E-05	0.004439	Cytochrome P450; contains IPR001128 (Cytochrome P450)
3.99588	6.45E-05	0.004524	Universal stress protein family protein; contains IPR014729 (Rossmann-like alpha/beta/alpha sandwich fold)
-3.99475	6.48E-05	0.004528	Sugar transporter, putative; contains IPR005828 (General substrate transporter), IPR016196 (Major facilitator superfamily domain, general substrate transporter)
3.992696	6.53E-05	0.004551	Shikimate kinase; contains IPR000623 (Shikimate kinase/Threonine synthase-like 1), IPR027417 (P-loop containing nucleoside triphosphate hydrolase)
-3.98612	6.72E-05	0.004661	Probable receptor-like protein kinase; contains IPR011009 (Protein kinase-like domain), IPR013320 (Concanavalin A-like lectin/glucanase, subgroup)
3.982638	6.82E-05	0.004712	Cytochrome P450, putative; contains IPR001128 (Cytochrome P450)
3.976051	7.01E-05	0.004826	Unknown protein
3.973536	7.08E-05	0.004859	UPF0503 protein At3g09070, chloroplastic; contains IPR008004 (Uncharacterised protein family UPF0503)
3.969015	7.22E-05	0.004934	Glutamyl-tRNA(Gln) amidotransferase subunit A; contains IPR000120 (Amidase), IPR023631 (Amidase signature domain)
3.966619	7.29E-05	0.004965	RNA binding protein; contains IPR012677 (Nucleotide-binding, alpha-beta plait)
3.964969	7.34E-05	0.004981	Unknown protein
3.961449	7.45E-05	0.005037	Homeobox-leucine zipper protein 9; contains IPR003106 (Leucine zipper, homeobox-associated), IPR009057 (Homeodomain-like)
3.958386	7.55E-05	0.00508	Auxin-induced SAUR-like protein; contains IPR003676 (Auxin responsive SAUR protein)
-3.95764	7.57E-05	0.00508	Proline rich protein; contains IPR016140 (Bifunctional inhibitor/plant lipid transfer protein/seed storage helical domain)
3.948906	7.85E-05	0.00525	Ribosomal RNA small subunit methyltransferase A; contains IPR001737 (Ribosomal RNA adenine methylase transferase)
-3.94453	8.00E-05	0.005289	Myb transcription factor; contains IPR009057 (Homeodomain-like)
3.945619	7.96E-05	0.005289	Fasciclin-like arabinogalactan protein family protein; contains IPR000782 (FAS1 domain)
3.944631	7.99E-05	0.005289	Desumoylating isopeptidase 2; contains IPR008580 (Domain of unknown function DUF862, eukaryotic)
-3.94283	8.05E-05	0.005308	Calmodulin-binding protein; contains IPR012442 (Protein of unknown function DUF1645)
3.936189	8.28E-05	0.005437	Transcription factor GRAS family protein; contains IPR005202 (Transcription factor GRAS)
-3.93222	8.42E-05	0.005508	Phosphatidylinositol N-acetyglucosaminlytransferase subunit P-like protein; contains IPR022212 (Protein of unknown function DUF3741), IPR025486 (Domain of unknown function DUF4378)
-3.92539	8.66E-05	0.005627	Leucine-rich repeat receptor-like serine/threonine-protein kinase; contains IPR001611 (Leucine-rich repeat)

3.925383	8.66E-05	0.005627	Zinc finger (Ran-binding) family protein; contains IPR001876 (Zinc finger, RanBP2-type)
-3.91443	9.06E-05	0.005868	Unusual floral organs; contains IPR001810 (F-box domain, cyclin-like)
3.900398	9.60E-05	0.006197	Centrin-2; contains IPR011992 (EF-hand-like domain)
3.895398	9.80E-05	0.006304	F-box family protein; contains IPR001810 (F-box domain, cyclin-like)
3.893895	9.86E-05	0.00631	1-aminocyclopropane-1-carboxylate oxidase 5; contains IPR005123 (Oxoglutarate/iron-dependent dioxygenase), IPR026992 (Non-haem dioxygenase N-terminal domain), IPR027443 (Isopenicillin N synthase-like)
-3.89263	9.92E-05	0.00631	Putative peptide/nitrate transporter; contains IPR000109 (Proton-dependent oligopeptide transporter family), IPR016196 (Major facilitator superfamily domain, general substrate transporter)
3.893253	9.89E-05	0.00631	Unknown protein
3.878235	0.000105	0.006672	Putative A-type response regulator 7; contains IPR011006 (CheY-like superfamily)
-3.86123	0.000113	0.00713	Transcription factor, putative; contains IPR005333 (Transcription factor, TCP)
-3.85856	0.000114	0.007183	Receptor-like protein kinase; contains IPR001611 (Leucine-rich repeat), IPR003591 (Leucine-rich repeat, typical subtype), IPR011009 (Protein kinase-like domain), IPR025875 (Leucine rich repeat 4)
3.853426	0.000116	0.007311	IQ domain-containing protein; contains IPR000048 (IQ motif, EF-hand binding site)
-3.85054	0.000118	0.007372	Mannose-6-phosphate isomerase; contains IPR001250 (Mannose-6-phosphate isomerase, type I)
3.844452	0.000121	0.007532	Putative uncharacterized protein At2g39855/T5I7.3
-3.83102	0.000128	0.007928	Protein trichome birefringence-like 33; contains IPR025846 (PMR5 N-terminal domain), IPR026057 (PC-Esterase)
-3.8205	0.000133	0.008219	WRKY transcription factor 10; contains IPR003657 (DNA-binding WRKY)
3.820886	0.000133	0.008219	Transcription factor; contains IPR011598 (Myc-type, basic helix-loop-helix (bHLH) domain)
-3.81943	0.000134	0.008227	Receptor-like protein kinase; contains IPR011009 (Protein kinase-like domain), IPR013210 (Leucine-rich repeat-containing N-terminal, type 2), IPR013320 (Concanavalin A-like lectin/glucanase, subgroup)
3.814829	0.000136	0.008354	LOB domain-containing protein, putative; contains IPR004883 (Lateral organ boundaries, LOB)
3.80979	0.000139	0.008498	SEC14 cytosolic factor family protein; contains IPR001251 (CRAL-TRIO domain), IPR011074 (CRAL/TRIO, N-terminal
3.800772	0.000144	0.008784	Early nodulin-like protein 1; contains IPR008972 (Cupredoxin)
-3.78951	0.000151	0.009161	Histidine kinase 2; contains IPR005467 (Signal transduction histidine kinase, core), IPR006189 (CHASE), IPR009082 (Signal transduction histidine kinase, homodimeric domain), IPR011006 (CheY-like superfamily)
3.788155	0.000152	0.009171	Calmodulin-binding protein-like protein; contains IPR012417 (Calmodulin-binding domain, plant)
3.787603	0.000152	0.009171	Unknown protein
-3.78215	0.000155	0.009344	Phantastica transcription factor a
3.781019	0.000156	0.009356	Pectin acetylesterase, putative; contains IPR004963 (Pectinacetylesterase)
3.775097	0.00016	0.00955	Unknown protein
3.762698	0.000168	0.010004	Zinc finger-homeodomain protein; contains IPR006456 (ZF-HD homeobox protein, Cys/His-rich dimerisation domain), IPR009057 (Homeodomain-like)

-3.7531	0.000175	0.010361	Alkaline alpha-galactosidase seed imbibition protein; contains IPR008811 (Raffinose synthase), IPR013785 (Aldolase-type TIM barrel)
3.746545	0.000179	0.010602	Transcription factor BIM1, putative; contains IPR011598 (Myc-type, basic helix-loop-helix (bHLH) domain)
3.728506	0.000193	0.011353	AT4g28240/F26K10_120; contains IPR022251 (Protein of unknown function wound-induced)
3.70188	0.000214	0.012574	Unknown protein; contains IPR022251 (Protein of unknown function wound-induced)
-3.67408	0.000239	0.01398	Transcription factor; contains IPR011598 (Myc-type, basic helix-loop-helix (bHLH) domain)
3.654078	0.000258	0.015069	Calcium-transporting ATPase 1; contains IPR001757 (Cation-transporting P-type ATPase), IPR023214 (HAD-like domain), IPR023298 (P-type ATPase, transmembrane domain), IPR024750 (Calcium-transporting P-type ATPase, N-terminal autoinhibitory domain)
3.644726	0.000268	0.015578	Auxin-induced protein-like protein; contains IPR003676 (Auxin responsive SAUR protein)
3.639525	0.000273	0.015846	Cytochrome P450, putative; contains IPR001128 (Cytochrome P450)
-3.62427	0.00029	0.016758	Unknown protein
3.623341	0.000291	0.016766	CM0545.320.nc protein
3.618292	0.000297	0.017043	Ring finger protein, putative; contains IPR013083 (Zinc finger, RING/FYVE/PHD-type)
3.596833	0.000322	0.018454	WRKY transcription factor 4; contains IPR003657 (DNA-binding WRKY)
3.589163	0.000332	0.018946	Auxin-responsive protein; contains IPR003311 (AUX/IAA protein)
3.585813	0.000336	0.019132	Probable receptor-like protein kinase; contains IPR011009 (Protein kinase-like domain), IPR013320 (Concanavalin A-like lectin/glucanase, subgroup)
3.579485	0.000344	0.019523	F-box family protein-like
3.578921	0.000345	0.019523	Neoxanthin synthase; contains IPR000253 (Forkhead-associated (FHA) domain), IPR025461 (Protein of unknown function DUF4281)
3.578034	0.000346	0.019529	Unknown protein
3.573797	0.000352	0.019787	Early response to dehydration 15-like protein; contains IPR009818 (Ataxin-2, C-terminal)
3.571738	0.000355	0.019883	Gibberellin 2-oxidase; contains IPR002283 (Isopenicillin N synthase), IPR026992 (Non-haem dioxygenase N-terminal domain), IPR027443 (Isopenicillin N synthase-like)
3.564701	0.000364	0.020362	Unknown protein
-3.56299	0.000367	0.020433	Multidrug resistance protein ABC transporter family; contains IPR011527 (ABC transporter, transmembrane domain, type 1), IPR027417 (P-loop containing nucleoside triphosphate hydrolase)
3.552527	0.000382	0.021198	Putative receptor kinase; contains IPR001611 (Leucine-rich repeat), IPR011009 (Protein kinase-like domain), IPR013210 (Leucine-rich repeat-containing N-terminal, type 2)
3.545736	0.000392	0.021687	Lipid transfer protein; contains IPR000528 (Plant lipid transfer protein/Par allergen), IPR016140 (Bifunctional inhibitor/plant lipid transfer protein/seed storage helical domain)
3.544301	0.000394	0.02174	Squamosa promoter binding protein; contains IPR004333 (Transcription factor, SBP-box)
3.53749	0.000404	0.022241	Multidrug resistance protein MdtK; contains IPR002528 (Multi antimicrobial extrusion protein)

3.529374	0.000417	0.022866	F8K7.26 protein; contains IPR008004 (Uncharacterised protein family UPF0503)
-3.52774	0.000419	0.02294	Unknown protein
3.526657	0.000421	0.022965	UDP-glycosyltransferase 1; contains IPR002213 (UDP-glucuronosyl/UDP-glucosyltransferase)
3.520499	0.000431	0.023436	Unknown protein
3.519481	0.000432	0.023456	Unknown protein
-3.51786	0.000435	0.023531	N-acetyltransferase, putative; contains IPR016181 (Acyl-CoA N-acyltransferase)
3.511882	0.000445	0.023996	Nucleoredoxin; contains IPR011424 (C1-like), IPR012336 (Thioredoxin-like fold)
3.505542	0.000456	0.024503	Transcription factor, putative; contains IPR011598 (Myc-type, basic helix-loop-helix (bHLH) domain)
3.500558	0.000464	0.02482	UDP-glucuronate 5-epimerase, putative; contains IPR001509 (NAD-dependent epimerase/dehydratase), IPR008089 (Nucleotide sugar epimerase)
3.500723	0.000464	0.02482	Zinc finger-homeodomain protein; contains IPR006456 (ZF-HD homeobox protein, Cys/His-rich dimerisation domain), IPR009057 (Homeodomain-like)
-3.4964	0.000472	0.025137	Gb|AAC95187.1; contains IPR021916 (Protein of unknown function DUF3527)
3.492127	0.000479	0.025469	Probable peptide/nitrate transporter; contains IPR000109 (Proton-dependent oligopeptide transporter family), IPR016196 (Major facilitator superfamily domain, general substrate transporter)
3.490257	0.000483	0.025574	AT5G28150-like protein; contains IPR008586 (Protein of unknown function DUF868, plant)
3.486017	0.00049	0.02589	Transcription factor; contains IPR011598 (Myc-type, basic helix-loop-helix (bHLH) domain)
-3.48544	0.000491	0.02589	Nucleic acid binding protein, putative; contains IPR007087 (Zinc finger, C2H2), IPR012317 (Poly(ADP-ribose) polymerase, catalytic domain)
3.482036	0.000498	0.02613	Cold induced protein-like
-3.48144	0.000499	0.02613	Lipid transfer protein-like; contains IPR016140 (Bifunctional inhibitor/plant lipid transfer protein/seed storage helical
-3.47593	0.000509	0.026597	Two-component response regulator-like protein; contains IPR009057 (Homeodomain-like)
3.46601	0.000528	0.027455	Putative RING-H2 finger protein; contains IPR010543 (Domain of unknown function DUF1117), IPR013083 (Zinc finger, RING/FYVE/PHD-type)
3.465881	0.000528	0.027455	Unknown protein
3.448783	0.000563	0.029171	F12M16.3
3.443912	0.000573	0.029618	Auxin response factor 5; contains IPR003311 (AUX/IAA protein), IPR010525 (Auxin response factor), IPR015300 (DNA-binding pseudobarrel domain)
-3.44192	0.000578	0.029754	Senescence-associated protein SAG102; contains IPR007650 (Protein of unknown function DUF581)
3.435508	0.000591	0.030046	Low-temperature-induced 65 kDa protein; contains IPR012418 (CAP160)
3.436125	0.00059	0.030046	MYB family transcription factor
3.437035	0.000588	0.030046	Early response to dehydration 15-like protein
3.436053	0.00059	0.030046	Unknown protein
-3.43554	0.000591	0.030046	Cyclin D3.2 protein; contains IPR013763 (Cyclin-like)

3.428954	0.000606	0.030696	Peroxidase 3, putative; contains IPR010255 (Haem peroxidase)
-3.42378	0.000618	0.031133	Cytochrome P450, putative; contains IPR001128 (Cytochrome P450)
3.423624	0.000618	0.031133	CLAVATA3/ESR (CLE)-related protein 25
3.420871	0.000624	0.031364	F-box family protein; contains IPR001810 (F-box domain, cyclin-like)
3.415923	0.000636	0.031852	Auxin-responsive protein; contains IPR003311 (AUX/IAA protein)
3.394771	0.000687	0.034324	Ice binding protein, putative; contains IPR008546 (Domain of unknown function DUF828), IPR013666 (Pleckstrin-like,
3.389904	0.000699	0.034751	F-actin capping protein beta subunit, putative; contains IPR001698 (WASH complex, F-actin capping protein, beta subunit)
-3.39055	0.000698	0.034751	ABC transporter G family member; contains IPR013525 (ABC-2 type transporter), IPR013581 (Plant PDR ABC transporter associated), IPR027417 (P-loop containing nucleoside triphosphate hydrolase)
-3.38394	0.000715	0.035264	Zinc finger protein; contains IPR013087 (Zinc finger C2H2-type/integrase DNA-binding domain)
3.384116	0.000714	0.035264	Unknown protein
3.383667	0.000715	0.035264	GATA transcription factor, putative; contains IPR013088 (Zinc finger, NHR/GATA-type)
3.380119	0.000725	0.035627	Dof zinc finger protein; contains IPR003851 (Zinc finger, Dof-type)
3.375996	0.000735	0.036068	Unknown protein
-3.364	0.000768	0.037573	Receptor kinase; contains IPR000225 (Armadillo), IPR001611 (Leucine-rich repeat), IPR003591 (Leucine-rich repeat, typical subtype), IPR011009 (Protein kinase-like domain), IPR013210 (Leucine-rich repeat-containing N-terminal, type 2), IPR025875 (Leucine rich repeat 4)
3.34542	0.000822	0.040077	Unknown protein
-3.34312	0.000828	0.040304	Response regulator; contains IPR011006 (CheY-like superfamily)
3.342101	0.000831	0.040345	Cation transport regulator-like protein; contains IPR006840 (ChaC-like protein), IPR013024 (Butirosin biosynthesis, BtrG-
3.336933	0.000847	0.040994	Unknown protein
3.326652	0.000879	0.042425	Charged multivesicular body protein 2a, putative; contains IPR005024 (Snf7)
-3.31708	0.00091	0.043679	DNA repair and recombination protein RAD54-like protein; contains IPR000330 (SNF2-related), IPR001650 (Helicase, C-terminal), IPR027417 (P-loop containing nucleoside triphosphate hydrolase)
-3.31707	0.00091	0.043679	Urease, alpha subunit; contains IPR002019 (Urease, beta subunit), IPR002026 (Urease, gamma/gamma-beta subunit), IPR005848 (Urease, alpha subunit), IPR008221 (Urease)
-3.31555	0.000915	0.043693	Inter-alpha-trypsin inhibitor heavy chain H3; contains IPR002035 (von Willebrand factor, type A), IPR013083 (Zinc finger, RING/FYVE/PHD-type)
3.315519	0.000915	0.043693	Xyloglucan endotransglucosylase/hydrolase 3; contains IPR008985 (Concanavalin A-like lectin/glucanases superfamily), IPR016455 (Xyloglucan endotransglucosylase/hydrolase)
-3.31297	0.000923	0.04398	HAD-superfamily hydrolase, subfamily IA, variant 3; contains IPR005833 (Haloacid dehalogenase/epoxide hydrolase), IPR006402 (HAD-superfamily hydrolase, subfamily IA, variant 3), IPR023214 (HAD-like domain)
-3.30687	0.000943	0.044833	Receptor-like protein kinase; contains IPR001611 (Leucine-rich repeat), IPR011009 (Protein kinase-like domain), IPR013210 (Leucine-rich repeat-containing N-terminal, type 2)

-3.30584	0.000947	0.044882	Response regulator 6; contains IPR011006 (CheY-like superfamily)
3.301298	0.000962	0.045497	Nematode-resistance protein; contains IPR009743 (Hs1pro-1, C-terminal), IPR009869 (Hs1pro-1, N-terminal)
3.295345	0.000983	0.046353	Acid beta-fructofuranosidase, putative; contains IPR001362 (Glycoside hydrolase, family 32), IPR008985 (Concanavalin A-like lectin/glucanases superfamily), IPR021792 (Beta-fructofuranosidase), IPR023296 (Glycosyl hydrolase, five-bladed beta-propellor domain)
3.288957	0.001006	0.047296	Unknown protein
-3.2871	0.001012	0.047488	Putative carboxyl-terminal proteinase; contains IPR004314 (Domain of unknown function DUF239), IPR025521 (Domain of unknown function DUF4409)
-3.28359	0.001025	0.047962	Universal stress protein family protein; contains IPR014729 (Rossmann-like alpha/beta/alpha sandwich fold)
3.281775	0.001032	0.048148	Putative calmodulin-related protein; contains IPR011992 (EF-hand-like domain)
3.2773	0.001048	0.048671	Ubiquitin carboxyl-terminal hydrolase; contains IPR001607 (Zinc finger, UBP-type), IPR011422 (BRCA1-associated 2), IPR013083 (Zinc finger, RING/FYVE/PHD-type)
3.277632	0.001047	0.048671	Cytokinin riboside 5'-monophosphate phosphoribohydrolase-like; contains IPR005269 (Cytokinin riboside 5'-monophosphate phosphoribohydrolase LOG)
3.275586	0.001054	0.048844	Basic helix-loop-helix protein, putative; contains IPR025610 (Transcription factor MYC/MYB N-terminal)
3.274015	0.00106	0.048993	Plant-specific domain TIGR01570 family protein; contains IPR006460 (Protein of unknown function DUF617, plant)
3.271848	0.001068	0.049246	Homogentisate 1,2-dioxygenase; contains IPR005708 (Homogentisate 1,2-dioxygenase), IPR014710 (RmlC-like jelly roll fold)

Supplemental Table S2.Candidate auxin-induced transcription factors in transcriptome

Gene ID	Log_2FC	*p* -value	Putative function in CuGenDB
Csa2G000030	0.501662	4.62E-06	Auxin response factor 1; contains IPR003311 (AUX/IAA protein), IPR010525 (Auxin response factor), IPR015300 (DNA-binding pseudobarrel domain)
Csa3G866510	1.056897	1.87E-19	Auxin response factor, putative; contains IPR003311 (AUX/IAA protein), IPR010525 (Auxin response factor), IPR015300 (DNA-binding pseudobarrel domain)
Csa1G042290	0.784998	1.75E-10	Ethylene-responsive transcription factor; contains IPR016177 (DNA-binding, integrase-type)
Csa5G598600	1.093711	6.91E-10	Ethylene-responsive transcription factor; contains IPR016177 (DNA-binding, integrase-type)
Csa7G432080	2.350764	1.16E-06	Ethylene-responsive transcription factor 7; contains IPR016177 (DNA-binding, integrase-type)
Csa4G652640	1.719131	3.92E-06	Ethylene-responsive transcription factor ; contains IPR016177 (DNA-binding, integrase-type)
Csa4G630010	0.603378	6.51E-06	Ethylene-responsive transcription factor 6; contains IPR016177 (DNA-binding, integrase-type)
Csa2G382550	0.710277	1.39E-05	Ethylene-responsive transcription factor ; contains IPR016177 (DNA-binding, integrase-type)

Supplemental Table S3. The analysis of CsESR2 promoter sequence from PlantCARE

Site Name	sequence	Position	Number	Function
TCA-element	CCATCTTTTT	-15	1	Salicylic acid-responsive element
TGA-element	AACGAC	-1120,-2811[a]	2	Auxin-responsive element
CGTCA-motif	CGTCA	-1865,-2676	2	MeJA-responsive element
AuxRE	TGTCTC	-2833	1	Auxin-responsive element
ABRE	ACGTG	-276[a],-1123[a], -1468[a],-1542, -2191,-2361	6	Abscisic acid-responsive element

Promoter sequence is based on 9930 V3.0. "a" indicates that the element exists in “-” strand of the promoter sequence.

Supplemental Table S4. Primer sequences used in this study

Name	Gene ID[a]	Forward Primer (5'-3')	Reverse Primer (3'-5')	Description
qCsa2G000030	Csa2G000030	GGCCTCTTGTTTCGTTACCTC	GGGTCATTTGTGCATACACCTC	*ARFs* in RNA-seq
qCsa3G866510	Csa3G866510	CCCCACAGACTAACCTTCTCG	CTGCCACCTGTTCACTATGTCC	
qCsa2G382550	Csa2G382550	TCCGAAATTCGTCACCCTTTA	GCAGTCAAATTGGCCGAGA	
qCsa4G630010	Csa4G630010	CGCTACAGAGGCGTCAGAAA	GGAAATCGGTTTGAAGAGGG	
qCsa4G652640	Csa4G652640	AGACAAAGGCATTGGGGTTC	TTGCACTTGGACTGTATGGAAA	*ERFs* in RNA-seq
qCsa7G432080	Csa7G432080	ATTGGGGCTCTTGGGTCTC	ACTGTTGGTCGTTCGGATTGT	
qCsa1G042290	Csa1G042290	TGATCCTGATGCCACTGACTACT	CTGCGTTCTTCTACTGCTTTCTT	
qCsESR2	Csa5G598600	GAGGACTCAAAGCTCGCACTAA	GGGTTCAGGGTAATCAAAGGTG	
qCsACO1	Csa6G160180	TTGAGGTACTGAACCACGGAAT	GGAGATGACGGAGGAAGAAAG	
qCsACO2	Csa6G511860	TGGACAAAGTGGAGAAGATGACAA	AGTTTCCACTGAATCCAACCCTT	
qCsACO3	Csa6G421630	TCTTAGCAAAGGCTTAGAGGC	TCACATAGCAGGTCCAACAAC	*ACO* family genes
qCsACO4	Csa4G361270	TAGCCTCACCGAATGGGAAC	CCTTTGGGAGGAACTTCTGCT	
qCsACO5	Csa2G000520	CATCCTCCTTCTCCAAGACG	CAGCCTGCTACCGTGTTTCT	
qCsACS1	Csa6G496450	GGGTCTTGCCGAGAATCAACTAT	GGGTAAGCCGTGGTAATCTTGG	
qCsACS2	Csa1G580750	CCTTACTATCCTGGATTTGACAGAG	AATGTCTTCGATTGTGGAGCGTTG	Sex determination genes
qCsACS11	Csa2G353460	GGAAGAGGAGATTGATGTTGGAGT	TTCAAAAGATGCCTCATATCCAC	
qCsWIP1	Csa4G290830	GTTGAGGCTGCCATGTT	GCAATGCCAAAGTTTCCC	
qCsACS4	Csa6G006800	AGCAGAAAATCAGCTTTCTCCCG	CAATGTCCTTGAAGTTCAGGAGACC	
qCsACS5	Csa4G099220	TGGGTCTGGCAGAAAATCAGTTATG	GCAGGCAAGCCATTGTAGTCTTG	
qCsACS6	Csa4G049610	TTAGCTGAAAATCAGCTTTCTTTTG	GAAGTTCGCTACAGCGTTTCTAAAC	ACS family genes
qCsACS8	Csa3G177920	GAGAACAGGCTGTGCTTTGATTTGC	CACAATCCATCGAAAGGCTGATAAG	
qCsACS9	Csa5G157380	GGATTTGAAACTGGCTGTAGCAAG	GAGTTTCGATTGCAGAGGTCAC	
qCsACTIN2	Csa6G484600	ATTCTTGCATCTCTAAGTACCTTCC	CCAACTAAAGGGAAATAACTCACC	Reference gene in qPCR / semi-qPCR
pM-P1	Csa2G353460	ACAACTATTGAAGTAAAAGATTCACT	CCCTACTCTCTTACCTAATCATTTT	
pM-P2	Csa2G353460	CTAATAATGTAGAGTTGTGGGGG	TTCTTTGTACTTAATTATGCAGACC	
pM-P3	Csa2G353460	AAGAAGGATGATTAGAGAAGTACAA	TCGAGGATATCTTCATGATGAA	Chip-qPCR
pM-P4	Csa2G353460	GGGTCTTAGGTTTAAGAGATAAGGA	TTTTAATTTTTTGCTAAAAAGGG	
pM-P5	Csa2G353460	CAAACACACCTACTCCTTTTCTTC	TCTTGGTATTTTCTTTTTAATACGG	
pM-P6	Csa2G353460	AATGATTGTTTTTTTCTTTTGGA	GGGAAAGAAAGAAGATGAAATAAT	
1305.4-CsESR2-6His	Csa5G598600	ACGGGGGACTCTAGAGGATCCATGGAAGAAGCACTAAGGCG	CTGGTCACCAATTCACACGTGTTAGTGGTGGTGGTGGTGGTGAGCA TTCTGTATTTTAGCAAC	Tobacco transient transformation
pBI121-pCsACS2	Csa2G353460	GACCATGATTACGCCAAGCTTGGGTTGGTGGGGTCATAAG	GGACTGACCACCCGGGGATCCTGATGTTTGTTCTTTTTTCCTTG	
AD-CsESR2	Csa5G598600	GTGGGCATCGATACGGGATCCATATGGAAGAAGCACTAAGGCG	ACGATTCATCTGCAGCTCGAGTTAAGCATTCTGTATTTTAGCAACA	
AD-Csa4G630010	Csa4G630010	GTGGGCATCGATACGGGATCCATATGGCTGCCGGAGACCG	ACGATTCATCTGCAGCTCGAGTCAGACAACTTCCACAGCTGG	
AD-Csa2G000030	Csa2G000030	GTGGGCATCGATACGGGATCCATATGAAAGCTCCCTCAAATGG	ACGATTCATCTGCAGCTCGAGTCATCGATTGAACGATGCAG	
pHis2-pCsACS1	Csa6M496450	GACTCACTATAGGGCGAATTCAGGGAAATTAGGTGTGTGTGTATAG	CGGATCGATTCGCGAACGCGTTTTTTCTTCGGTTGTGCTGC	
pHis2-pCsACS2	Csa2G353460	GACTCACTATAGGGCGAATTCGGGTTGGTGGGGTCATAAG	CGGATCGATTCGCGAACGCGTTGATGTTTGTTCTTTTTTCCTTG	Yeast One-hybrid system
pHis2-pCsACS11	Csa2M353460	GACTCACTATAGGGCGAATTCGAGATGTAAAGGCGTCATTTGAG	CGGATCGATTCGCGAACGCGTCATATGTGAGTGTACAGTAATATG	
pHis2-pCsACO2	Csa6G511860	GACTCACTATAGGGCGAATTCATTGATTGATTAACCACATTTCT	CGGATCGATTCGCGAACGCGTTGTTTCTCTCTAGAACTTATTGTA	
pHis2-pCsWIP1	Csa4M290830	GACTCACTATAGGGCGAATTCCTATTATTATTGTTATTGGTTG	CGGATCGATTCGCGAACGCGTCTTTTTTTTTCTTTTGATGG	
CsESR2-SP6	Csa5G598600	GATTTAGGTGACACTATAGAATGCTATGGAAGAAGCACTAAGGCG	——	*In Situ* Hybridization
CsESR2-T7	Csa5G598600	TGTAATACGACTCACTATAGGGAGCATTCTGTATTTTAGCAACATAA	——	
pGreenII62-SK-CsESR2	Csa5G598600	AGTGGATCCCCCGGGCTGCAGATGGAAGAAGCACTAAGGCG	GGTACCGGGCCCCCCCTCGAGTTAAGCATTCTGTATTTTAGCAACA	Dual-luciferase reporter assay system
pGreenII0800-pCsACS2	Csa2G353460	GGTACCGGGCCCCCCCTCGAGGGGTTGGTGGGGTCATAAG	AGTGGATCCCCCGGGCTGCAGTGATGTTTGTTCTTTTTTCCTTG	
pGreenII-35S-CsESR2	Csa5G598600	GCCCAAGCTACGCGTCTCGAG ATGGCTGCCGGAGACCG	TCCCCCGGGCTGCAGGAATTCAGCATTCTGTATTTTAGCAACATAA	Subcellular localization
CsACS11-Check	Csa2G353460	ATGGCATCCTTGTCTTCTAAAGC	TACGTTAGTGGGGGAGAGTTCAT	Check for 406an mutant

[a] The gene ID is based on 9930 V2.0.
